# Proteomics of *Campylobacter jejuni* Growth in Deoxycholate Reveals Cj0025c as a Cystine Transport Protein Required for Wild-type Human Infection Phenotypes

**DOI:** 10.1074/mcp.RA120.002029

**Published:** 2020-11-23

**Authors:** Lok Man, Ashleigh L. Dale, William P. Klare, Joel A. Cain, Zeynep Sumer-Bayraktar, Paula Niewold, Nestor Solis, Stuart J. Cordwell

**Affiliations:** 1 School of Life and Environmental Sciences, The University of Sydney, Australia; 2 Charles Perkins Centre, The University of Sydney, Australia; 3 Discipline of Pathology, School of Medical Sciences, The University of Sydney, Australia; 4 Sydney Mass Spectrometry, The University of Sydney, Australia

**Keywords:** Bacteria, Virulence, Metabolomics, Mass Spectrometry, Pathogens, Bacterial pathogenesis, Campylobacter jejuni, Cystine, Nutrient transport, Sulfur

## Abstract

*Campylobacter jejuni* is a major cause of food-borne gastroenteritis. Proteomics by label-based two-dimensional liquid chromatography coupled to tandem mass spectrometry (LC-MS/MS) identified proteins associated with growth in 0.1% sodium deoxycholate (DOC, a component of gut bile salts), and system-wide validation was performed by data-independent acquisition (DIA-SWATH-MS). LC-MS/MS quantified 1326 proteins (∼82% of the predicted *C. jejuni* proteome), of which 1104 were validated in additional biological replicates by DIA-SWATH-MS. DOC resulted in a profound proteome shift with 512 proteins showing significantly altered abundance. Induced proteins were associated with flagellar motility and antibiotic resistance; and these correlated with increased DOC motility and resistance to polymyxin B and ciprofloxacin. DOC also increased human Caco-2 cell adherence and invasion. Abundances of proteins involved in nutrient transport were altered by DOC and aligned with intracellular changes to their respective carbon sources. DOC increased intracellular levels of sulfur-containing amino acids (cysteine and methionine) and the dipeptide cystine (Cys-Cys), which also correlated with reduced resistance to oxidative stress. A DOC induced transport protein was Cj0025c, which has sequence similarity to bacterial Cys-Cys transporters. Deletion of *cj0025c* (Δ*cj0025c*) resulted in proteome changes consistent with sulfur starvation, as well as attenuated invasion, reduced motility, atypical morphology, increased antimicrobial susceptibility and poor biofilm formation. Targeted metabolomics showed Δ*cj0025c* could use known *C. jejuni* amino and organic acid substrates commensurate with wild-type. Medium Cys-Cys levels however, were maintained in Δ*cj0025c* relative to wild-type. A toxic Cys-Cys mimic (selenocystine) inhibited wild-type growth, but not Δ*cj0025c*. Provision of an alternate sulfur source (2 mm thiosulfate) restored Δ*cj0025c* motility. Our data confirm that Cj0025c is a Cys-Cys transporter that we have named TcyP consistent with the nomenclature of homologous proteins in other species.

## Teaser

Growth in the bile salt component deoxycholate (DOC) results in a profound change in the *Campylobacter jejuni* proteome that is reflected in phenotypic alterations including increased virulence, antibiotic resistance, and nutrient transport. A significantly induced protein was Cj0025c, which shares sequence similarity with TcyP cystine transporters from other bacteria. We show that Cj0025c is required for cystine uptake and deletion of *cj0025c* results in a sulfur-starved proteome and loss of wild-type phenotypes including human cell invasion, motility and helical morphology.

## Graphical Abstract

**Figure:**
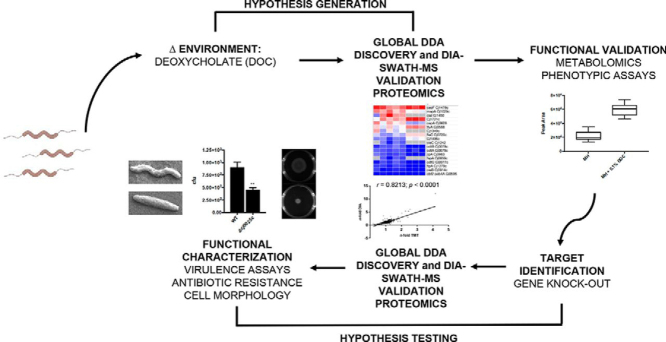

**Highlights**

• Growth in the bile salt deoxycholate (DOC) induces virulence proteins in *C. jejuni*.
• A putative symporter Cj0025c is associated with DOC growth and cystine transport.
• Deletion of *cj0025c* results in loss of cystine transport and a sulfur starved proteotype.
• Cj0025c is required for wild-type virulence phenotypes including human cell invasion.

*Campylobacter jejuni* is a Gram-negative, helical, and microaerophilic bacterium with polar flagella that confer motility. The organism is a common cause of food- and water-borne disease (1) and human infections are generally associated with consumption of poorly prepared poultry, because *C. jejuni* is largely considered a commensal in avian species, although this concept has also been challenged (2, 3, 4). Human disease often requires only low numbers of infecting cells (500–800 cells; a lower infective dose than *Salmonella* or pathogenic *E. coli* (5)) and symptoms can range from mild, non-inflammatory diarrhea through to abdominal cramps, vomiting and inflammation. *C. jejuni* is self-limiting in most clinical cases but has been linked to the development of Guillain-Barré Syndrome (GBS), an acute, debilitating immune-mediated disorder of the peripheral nervous system. Approximately 40% of GBS patients show serological evidence of a prior *C. jejuni* infection (6).

The “human-chicken paradigm” of why *C. jejuni* causes disease in humans but not poultry remains poorly understood. Colonization in both hosts has been linked with several *C. jejuni* traits, of which flagellar motility appears to be the most crucial (7, 8). Weakly motile *C. jejuni* are poor chicken colonizers and attenuated for human intestinal epithelial cell adherence and invasion. Many other factors, including chemotaxis and protein glycosylation, are also related to infection of both hosts (9, 10, 11, 12). Human pathogenesis involves adherence to epithelial cells, intracellular invasion, and toxin production (13). Colonization of chickens and human epithelia is facilitated by the fibronectin (Fn)-binding proteins CadF (14, 15) and FlpA (16), and many other ‘virulence’ factors have been described, including the surface-exposed glycoprotein adhesin JlpA (17, 18) and the PEB antigens (19, 20). Invasion and cytotoxicity appear largely mediated by the secreted *Campylobacter* invasion antigens (CiaBC, flagellin-like FlaC, FspA (21, 22, 23)) and the “cytolethal distending” toxin subunits (CdtABC; (24)).

*C. jejuni* can persist in a range of diverse and potentially stress-inducing environments, including different temperatures, osmolarities, iron and other trace element limitation, presence of toxic compounds and diverse nutrient availabilities. *C. jejuni* grows preferentially between 37–42 °C (human and poultry body temperatures) but can survive at temperature extremes (*e.g.* 4 °C; facilitating contamination of >75% of supermarket chicken products (25, 26)). The ability of *C. jejuni* to utilize host-specific nutrients is considered a major factor in infection. *C. jejuni* is largely thought to be asaccharolytic, although some strains can catabolize fucose (27). Amino and organic acids (28, 29, 30) are primary carbon sources and *C. jejuni* preferentially utilizes (in order) serine (Ser), aspartate (Asp), glutamate (Glu) and proline (Pro) (31). Some strains possess a γ-glutamyltranspeptidase (GGT) that enables glutamine (Gln) use (32, 33), whereas others contain an asparaginase (AnsB) that converts extracellular asparagine (Asn) to Asp (32). Ser uptake is mediated by the SdaC transporter and is converted to pyruvate *via* the l-Ser dehydratase SdaA (34, 35). Pro is the least preferred amino acid and following uptake by the PutP transporter (35) is converted to Glu *via* 1-pyrroline-5-carboxylate (catalyzed by PutA). Glu and Asp uptake are mediated by several transporters including those encoded by the PEB locus and the C_4_-dicarboxylate transporters, DcuA, DcuB and DctA, which also transport fumarate and succinate (36). Other organic acids catabolized by *C. jejuni* include malate, pyruvate and acetate (for which no specific transporters have been identified), citrate (likely transported by the Cj0203 citrate transporter homolog), lactate and α-ketoglutarate (KG), which are transported by the LctP and KgtP permeases, respectively (37).

Sulfur is an important element required for cysteine (Cys) and methionine (Met) biosynthesis, and for the formation of Fe-S clusters in proteins such as SdaA, ferredoxin, Nap nitrate reductase and aconitase (AcnB). *C. jejuni* is auxotrophic for Cys, despite being unable to utilize Cys as a sole carbon source, and this phenotype cannot be rescued by addition of sulfate as a sulfur source (38). *C. jejuni* lacks several enzymes in the assimilatory sulfur biosynthesis pathway, as well as typical bacterial ABC-type transport proteins specific for sulfate (38). Although sulfate cannot be used, alternative sulfur sources, including hydrogen sulfide and thiosulfate, allow *C. jejuni* growth (38, 39). Exogenous glutathione (GSH, a Glu-Cys-Gly tripeptide), can be acted upon by GGT-producing strains to produce Glu as a carbon source and Cys-Gly that can be imported by the *Campylobacter* peptide transporter CptA (33, 38) as a source of Cys. Additional dipeptides that provide alternative Cys sources include Gly-Cys and Glu-Cys, however transporters for these are currently unknown, and additional Cys-containing dipeptides, including cystine (Cys-Cys) have not, to our knowledge, been tested.

Here we performed proteomics of the *C. jejuni* response to growth in the presence of the bile salt constituent 0.1% sodium deoxycholate (DOC) using a label-based LC-MS/MS discovery set and global validation by data-independent acquisition (DIA-SWATH-MS). Nutrient transporters were significantly altered by growth in DOC, consistent with *in vivo* triggers for colonization requiring specific carbon sources. DOC induced a virulence-associated phenotype by increasing motility, antibiotic resistance, and adherence and invasion of human epithelial cells. The product of the *cj0025c* gene was significantly induced by DOC. Multi-omics and functional analyses confirmed that Cj0025c is a Cys-Cys transporter, required for several wild-type virulence attributes including motility, biofilm formation and cell invasion.

## EXPERIMENTAL PROCEDURES

##### Experimental Design and Statistical Rationale

For discovery-based quantitative proteomics by tandem mass tag (TMT) labeling and LC-MS/MS of *C. jejuni* growth in 0.1% DOC, 4 biological replicates (control and DOC growth) were processed in tandem. Two additional biological replicates were generated for the DIA-SWATH-MS validation set. Analysis of *C. jejuni* NCTC11168 wild-type (WT) compared with a Δ*cj0025c* mutant were carried out as above, except 5 biological replicates were analyzed by label-based discovery. Samples for the 2 technical approaches were processed independently of each other. Prior to TMT labeling, equal aliquots from each biological replicate were split to form an internal technical replicate. For targeted metabolomics, 3 biological replicates were prepared in triplicate extractions (9 replicates in total). MS data have been deposited to the ProteomeXchange Consortium via PRIDE with the data set identifier PXD017934.

##### Bacterial Strains and Growth Conditions

*C. jejuni* NCTC11168 (WT) and NCTC11168 Δ*cj0025c* (*cj0025c* deleted in NCTC11168 and DNA were sourced from the *Campylobacter* Resource Facility and provided as a gift by Prof. Brendan Wren) were prepared and grown as previously described (40). Δ*cj0025c* was grown in the presence of kanamycin (*K_m_*; 30 μg/ml) for selection, and 25 μg/ml trimethoprim (all antibiotics Sigma, St. Louis, MO) used for all strains when grown in Mueller-Hinton (MH) medium. Briefly, cells were grown from glycerol stocks in MH broth (Oxoid, Basingstoke, UK) for 48 h at 37 °C under microaerophilic conditions (5% O_2_, 10% CO_2_, 85% N_2_). Cells were sub-cultured into fresh MH broth (in the presence or absence of 0.1% DOC; Sigma) at an initial OD_600_ of 0.1 and grown until late exponential phase. Cells were collected by centrifugation before lyophilization. For culture supernatant levels of *C. jejuni* WT and Δ*cj0025c* amino acids, organic acids and sulfur-containing amino acids, cells were also grown in defined MCLMAN medium, supplemented with 10 mm carbon sources and/or 0.8 mm cystine (41).

##### Western Blotting

Proteins were transferred from SDS-PAGE gels to polyvinylidenedifluoride (PVDF) membrane for 1 h at 400 mA (4^°^C). Membranes were blocked overnight at 4^°^C in 5% bovine serum albumin (BSA) in 20 mm Tris, 150 mm NaCl, 0.1% Tween-20, pH 7.4. Membranes were probed with either a 1:1000 CadF-specific or a 1:1000 JlpA-specific rabbit anti-serum overnight at 4^°^C (18, 42). Proteins were detected using a 1:1000 dilution of goat-anti-rabbit immunoglobulin, followed by incubation with Supersignal^TM^ West Pico Chemiluminescent substrate according to the manufacturer's instructions (Thermo Scientific, Waltham MA) and visualized using a ChemiDOC^TM^ MP Imaging System (Bio-Rad, Hercules CA).

##### Quantitative PCR (qPCR)

Quantitative PCR (qPCR) was performed as previously described (12). Primers for *cj0025c* were as follows: forward 5′-CTAATGCTTTCACAAGCTCA-3′ and reverse 5′-TCCTGAGACATTGCTTGCTG-3′. All reactions were performed in technical duplicate. Quantification of gene expression was performed using the 2^ΔΔ^CT method, on a total of *n* = 3 biological replicates with 16S primers as *per* (12) used as the housekeeper gene.

##### Phenotypic Assays

Motility was assessed as described previously (40) using semi-solid MH medium with 0.4% agar. Plates were inoculated with 2 μl of an overnight biphasic culture (OD_600_ 0.5) and incubated for 48 h at 37 °C under microaerophilic conditions and motility measured by diameter of bacterial spread. For testing thiosulfate (TS) effects, 2 mm TS (Sigma) was added to the medium. Antibiotic sensitivities were measured using Etest® strips for polymyxin B, ciprofloxacin, erythromycin, and amoxacillin/clavulanic acid (Biomérieux, France). A 1 ml culture grown as above was spread on MH agar plates and an Etest® strip added to the center. Plates were incubated for 48 h at 37 °C under microaerophillic conditions. Resistance against oxidative stress was measured by exposure to 5 mm H_2_O_2_ for 30 min (12). Cultures were serially diluted and spread onto agar plates, incubated for 48 h at 37 °C under microaerophilic conditions and colony forming units (cfu) enumerated. Biofilm formation was assessed as *per* (12) with minor modifications; briefly, overnight cultures were diluted to an OD_600_ of 0.1 in 24 well, flat-bottom cell culture plates containing fresh Brain-Heart Infusion (BHI; Oxoid) medium or BHI supplemented with 5% (v/v) chicken exudate (‘juice’; (43)). Plates were incubated for 48 h under microaerophilic conditions. Non-adherent, non-biofilm cells were removed by washing with 1 ml of sterile PBS. 1.2 ml of fresh medium supplemented with 0.01% (w/v) 2,3,5-triphenyltetrazolium chloride (TTC) was added to each well and further incubated at 37 °C under micraerophilic conditions for 72 h. TTC was removed and the wells air dried. Bound dye was dissolved using 20% acetone/80% ethanol and the absorbance A_500_ of the solution measured. Human Caco-2 cell adherence and invasion assays were performed using the gentamicin protection method as described (12). Data are from *n* = 6 replicates.

##### Selenocystine Inhibition Assay

Selenocystine (Se-Cys-Cys) inhibition assay was performed as described (44). Briefly, cultures were normalized to OD_600_ of 1 and spread across MCLMAN agar to generate a bacterial lawn. After being allowed to dry, a sterile 10 mm diameter paper disk was placed in the center and 10 μl of 50 mm Se-Cys-Cys (Sigma) added to the disk. Plates were incubated under microaerophilic conditions for 48 h and the zone of inhibition measured.

##### Lipid A Analysis

Lipid A extraction was performed as *per* (45). Briefly, cells were solubilized in 4:3 70% isobutyric acid/1 M ammonium hydroxide (Sigma) and boiled at 100 °C for 30 min. Insoluble material was removed by centrifugation for 15 min at 2000 × *g*, following which supernatants were diluted 1:1 with ultrapure water and lyophilized. Extracts were washed with methanol twice before being reconstituted in 2:1:0.25 chloroform/methanol/water. Aliquots were spotted directly onto stainless steel targets 1:1 with the matrix norharmane (10 mg/ml [Sigma] in 2:1:0.25 chlorofom/methanol/water). Mass spectra were generated on a Bruker UltrafleXtreme (Bruker Daltonics, Billerica, MA) operated in reflectron mode across a mass range of 1000 to 2500 *m/z*, and a laser frequency of 2000 Hz using 40% global intensity.

##### Scanning Electron Microscopy (S.E.)

Cells were transferred to 12 well culture plates and allowed to settle onto Nunc Thermanox™ coverslips (Thermo Scientific) for 2 h. Cells were gently washed with 1× phosphate-buffered saline (PBS) 3 times, fixed with 2.5% glutaraldehyde for 1 h and then post-fixed in 1% osmium tetroxide (Sigma), dehydrated with hexamethyldisilazane, mounted on S.E. imaging stubs, and finally sputter coated with 1.5 nm gold. Cells were imaged with a Zeiss Sigma high definition field emission gun (HD-FEG) S.E. microscope at 10,000 × magnification.

##### Targeted Metabolomics by LC-MS/MS

Metabolites and MS parameters (parent, product ion *m/z* [transitions], retention time, collision energy and declustering potential) are summarized in supplemental Table S1. Cells (*n* = 9 samples from 3 biological replicates) were lysed in ultrapure water by 6 rounds of 30 s beadbeating. For assay of extracellular metabolites, aliquots from culture supernatants were collected at 0, 4, 24, 48 and 72 h growth and filtered through 0.22 μm polyethersulfone (PES) filters, following collection of intact cells by centrifugation, and stored at −30 °C. 25 μl of each sample was added to 75 μl of extraction buffer (0.1% formic acid in 80:20 ethanol/water (v/v)), mixed with vortexing, and incubated at 4 °C for 2 h. Solutions were vortexed then centrifuged at 14,000 × *g* at 4 °C for 15 min. 50 μl of supernatant was lyophilized and metabolites were resuspended in HPLC-grade water, and diluted as necessary. LC-MS/MS was performed as *per* (12, 46). Metabolites were loaded onto either a Luna Phenyl-Hexyl column (50 mm × 1 mm x 5 μm particle size) (Phenomenex, Torrance CA) or a Synergi Polar-RP (reversed phase), 80 Å column (50 mm × 1 mm × 4 μm particle size) (Phenomenex) using a Nexera UHPLC system (Shimadzu, Kyoto, Japan). Loading and elution were as previously described (12). Metabolites were eluted into a Q-TRAP 5500 mass spectrometer (SCIEX, Framingham MA) operated in targeted MRM mode. Data files were imported into Skyline (v. 4.1.0.18169), and peak areas manually integrated. Statistical analysis was performed in Metaboanalyst (v.4.0) (47).

##### Quantitative Proteomics by LC-MS/MS

Peptides were prepared as described previously (12) from cells reconstituted in 100 mm HEPES, pH 7.5 and lysed by 6 rounds of 30 s beadbeating. Proteins were precipitated in 1.8:2:2 water/methanol/chloroform, collected by centrifugation, washed twice with methanol, and solubilized in 8 m guanidine-HCl, 100 mm HEPES, pH 7.6. Reduction and alkylation were performed with 10 mm dithiothreitol (DTT) and 20 mm iodoacetamide (IAA) for 1 h each, respectively. Proteins were digested with sequencing grade modified trypsin (Promega, Madison WI), at a protein/protease ratio of 30:1 overnight at 37 °C. Peptides were desalted by solid phase extraction using hydrophilic-lipophilic balance (HLB) cartridges (Waters, Bedford MA) according to the manufacturer's instructions. After this step, samples for DIA-SWATH-MS were lyophilized (see below). For label-based analysis, samples were labeled with 6-plex TMT (Thermo Scientific) according to the manufacturer's instructions. Excess label was quenched with hydroxylamine hydrochloride, after which samples were pooled and desalted using HLB cartridges, as above. TMT labeled samples and pooled, unlabeled samples used to generate SWATH libraries (see below), were fractionated offline by hydrophilic interaction liquid chromatography (HILIC) prior to LC-MS/MS. Samples were reconstituted in 90% acetonitrile (MeCN), 0.1% trifluoroacetic acid (TFA) and loaded onto a 20 cm × 320 μm column packed in-house with 3 μm TSK-amide 80 particles (TOSOH Bioscience, King of Prussia PA) in 100% buffer B (90% MeCN, 0.1% TFA) at 10 μl/min for 10 min using an Agilent 1200 LC system (Agilent Technologies, Santa Clara, CA). Peptides were eluted by adjusting the mobile phase to 70% buffer A (0.1% TFA) over a 40 min linear gradient at 6 μl/min, and collected in 1 min fractions. Adjoining fractions were pooled to a final 6–12 fractions and lyophilized. TMT labeled peptide fractions were separated by reversed phase chromatography over a 90 min gradient as described (12) into a Q Exactive™ HF Hybrid Quadrupole Mass Spectrometer (QE-HF; Thermo Scientific). The QE-HF was configured to perform one full scan MS experiment (scan range 300 to 1650 *m/z*, resolution of 60000, automatic gain control [AGC] of 3e6 and a maximum ion injection time [IT] of 50 msec) with the top 15 precursors in fulfilment of the selection criteria (charge state 2–4, minimum intensity greater than 9.2e4, dynamic exclusion window of 40 s) selected for MS/MS (scan range 200 to 2000 *m/z* with a fixed first mass of 110 *m/z*, resolution of 15,000, AGC of 1e6, maximum IT of 50 msec, isolation window 1.4 *m/z*, normalized collision energy [NCE] set as 29).

##### Processing of Mass Spectrometry Files

Data files from TMT experiments were processed in Proteome Discoverer (v. 2.2; Thermo Scientific) and searched against the UniProt *C. jejuni* NCTC11168 genome database (UP000000799; organism ID 192222; release May 24, 2018 last modification; 1623 proteins (48)) with the SequestHT algorithm. Search parameters included full-trypsin digest with a maximum 2 missed cleavages and carbamidomethyl (C) as a fixed modification; and with variable modifications, oxidation (M), TMT-6plex (peptide *N*-term, K), and using precursor and fragment ion tolerances of 20 ppm. Data analysis was performed as *per* (12) with minor modifications. Briefly, peptide level false discovery rate (FDR) was established using Percolator (v. 2.08.01). Rank 1 peptide spectral matches (PSMs) conforming to this 1% peptide FDR were exported, and reporter intensities normalized to the total reporter ion intensity across all channels. Peptides with ambiguous protein assignments were removed from further analysis. Reporter signals for included PSMs were averaged to yield peptide-level quantitation and data from peptides representing each protein were averaged to determine protein-level quantitation. Proteins with a minimum of 2 unique identified peptides were imported into MetaboAnalyst (v 4.0) for statistical analysis by one sample *t*-tests performed on the log_2_ transformed protein-level ratios from all available biological replicates. Using the settings unpaired analysis and equal variance, statistical significance was determined using a *p* value cut-off set to *p* = 0.05. A minimum protein difference corresponding to a log_2_ fold change of ±(0.6) (∼±1.5-fold; 0.1% DOC) and ±(0.5) (∼1.4-fold; Δ*cj0025c*) was required for a protein to be considered as altered in abundance, and the values for all proteins in each set were arrayed by volcano plot to highlight those statistically significantly changing. For further stringency, proteins with fold-change values close to the cut-off were only included when validation by DIA-SWATH-MS was available. Data were averaged from all biological replicates in which quantitation was achieved (>2 peptides) and proteins were included only if quantified in a minimum of 2/4 (0.1% DOC) and 2/5 (Δ*cj0025c*) biological replicates. Missing values were not imputed. All peptide identifications are contained within Supplementary Data files 11168DOC_Peptide and 11168cj0025c_Peptide.

##### Validation by DIA-SWATH-MS

DIA-SWATH-MS was performed as *per* (12). Briefly, unlabeled peptides were reconstituted in 3% MeCN, 1% FA and loaded onto a 40 cm × 75 μm pulled column packed in-house with 3 μm ReproSil-Pur 120 C_18_-AQ material maintained at a column temperature of 45 °C at 200 nL/min in 98% buffer A (1% FA) using an Ekspert 425 nanoLC system (SCIEX). Peptides were eluted by altering the mobile phase to 28% buffer B (80% MeCN, 1% FA) over a 146 min linear gradient into a TripleTOF® 6600 mass spectrometer (SCIEX). For generation of the *C. jejuni* NCTC11168 spectral library, duplicate biological preparations of peptides from the WT controls were analyzed. The 6600 was configured to perform one full scan MS experiment (scan range 350 to 1500 *m/z*, accumulation time 50 msec) with a maximum of 80 precursors in fulfilment of the selection criteria (charge state 2–5, intensity >400, dynamic exclusion window of 20 s) selected for MS/MS in high sensitivity mode (scan range 100–1500 *m/z*, dynamic collision energy [DCE] set as true, mass tolerance of 20 ppm). For DIA-SWATH-MS, the instrument was configured to perform one full scan MS experiment (scan range 350 to 1500 *m/z*, accumulation time 50 msec) followed by 80 variable SWATH windows over the mass range 350 to 1500 *m/z* (accumulation time 25 msec). Window size was determined using the SWATH variable window calculator tool (SCIEX) from summed total ion chromatograms (TIC) of library-generated samples. SWATH library data files were processed in Protein Pilot (v. 5.0) and searched against the UniProt *C. jejuni* NCTC11168 proteome (as above) using the Paragon algorithm. Search parameters were sample set as identification; trypsin digestion, maximum 2 missed cleavages; Cys alkylation, IAA; instrument, TripleTOF 6600; search effort as thorough ID, and using a detected protein threshold of 0.05. The group file was then imported into PeakView (v. 2.2.0.11391) using a total protein number corresponding to a global protein level FDR of 1%. SWATH files were processed against the resulting library using the following settings; a peptide confidence threshold of 95%, 1% FDR, a minimum of 6 peaks *per* peptide selected for highest intensity across a retention time tolerance of 4 min, and an extracted ion chromatogram (XIC) width/mass tolerance of 20 ppm. Acquisitions were aligned by manually selecting a set of high-scoring peptides and generating a retention time standard curve in Peakview that was then applied to all samples. Peptide areas were summed to yield a total protein area that was used for relative quantitation.

## RESULTS

##### DOC Induces a C. jejuni Proteome Associated with Altered Virulence and In Vivo Growth

**Fig. 1:**
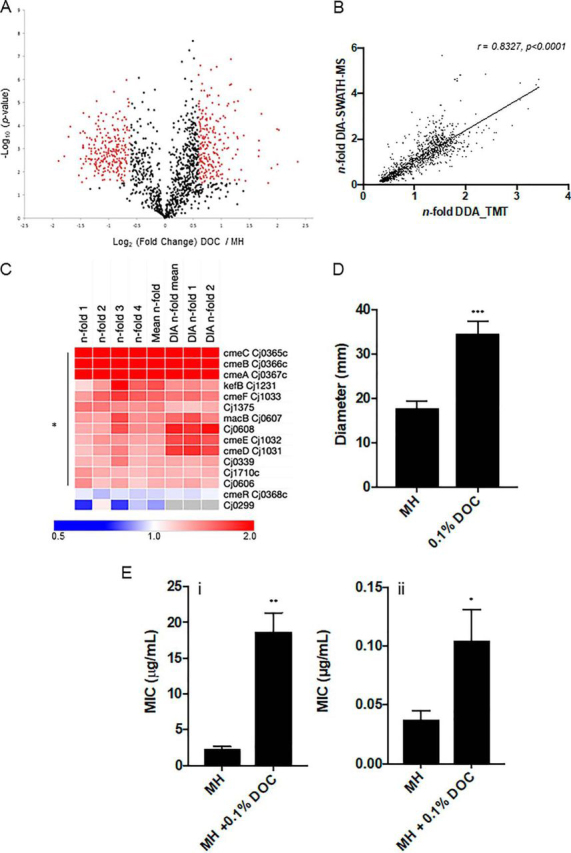
**DOC induces a large proteome shift in *C. jejuni* NCTC11168 and influences phenotypes associated with virulence.***A*, Volcano plot for cells grown in 0.1% DOC compared with control; *x* axis represents averaged Log_2_(DOC/control), *y* axis represents log_10_(*p* value). Significantly differentially abundant proteins are highlighted in red (*p* < 0.05); *B*, Correlation plot based on *n*-fold changes observed in the discovery set (*n*-fold DDA_TMT) and validation set (*n*-fold DIA-SWATH-MS), Pearson correlation *r* = 0.8213 (*p* < 0.0001) determined from 1104 aligned proteins; *C*, Heat map of *C. jejuni* proteins associated with antibiotic resistance and efflux ordered by largest mean *n*-fold change (label-based discovery; top). Data from each of 4 label-based replicates (*n*-fold 1–4) and DIA-SWATH-MS validation (‘DIA’ mean of 2 biological replicates [DIA *n*-fold 1–2]) are shown. Values are gray where the protein was not identified by DIA-SWATH-MS (* denotes proteins significantly altered in abundance); *D*, Increased motility associated with growth in 0.1% DOC based on colony diameter in mm on semi-solid agar (*** *p* < 0.001); *E*, Antibiotic resistance assays for (i) polymyxin B, (ii) ciprofloxacin (** *p* < 0.005, * *p* < 0.05).

Label-based quantitative proteomics post-trypsin digest was applied to identify *C. jejuni* NCTC11168 proteins associated with growth in 0.1% DOC. TMT labeling and LC-MS/MS provided a “discovery” data set that identified and quantified 1326 proteins (with ≥ 2 peptides *per* protein) from a minimum of 2/4 biological replicates and which represent 81.7% of the *C. jejuni* NCTC11168 predicted proteome encoded by 1623 genes (Fig. 1*A*, supplemental Data S1). System-wide validation was performed using additional biological replicates that were subjected to trypsin digest and DIA-SWATH-MS, which enabled the independent relative quantitation of 1112 proteins (representing 68.5% of the predicted proteome; supplemental Data S1), with 8 identified in the validation set alone (1104 proteins therefore identified in both discovery and validation cohorts). The label-based and DIA-SWATH-MS data sets showed a Pearson correlation of *r* = 0.8327 (Fig. 1*B*), indicating strong alignment between the discovery and validation approaches. Log_2_ (fold change) data were converted to *n*-fold change for subsequent visualization and analysis and the complete data sets are shown in supplemental Fig. S1.

0.1% DOC induced a profound shift in the *C. jejuni* proteome with 512 proteins (38.6% of the total identified proteome) considered altered in abundance (244 present at significantly induced, and 268 at significantly reduced, abundance). Two proteins (Cj0879c and Cj1171c) with a conflicting fold change as determined by DIA-SWATH-MS compared with label-based analysis (opposite change in abundance or no change in abundance) were removed. We employed STRINGdb, which identified 3 major functional clusters in the up-regulated protein set (supplemental Fig. S2A); antibiotic resistance (Fig. 1*C*), flagellar motility and nutrient acquisition. We validated these data by conducting motility and antibiotic resistance assays in the presence or absence of 0.1% DOC. DOC resulted in an ∼2-fold increase in *C. jejuni* motility (Fig. 1*D*) and significantly increased resistance to the antimicrobial peptide polymyxin B (8.01-fold, *p* < 0.005) and the fluoroquinolone, ciprofloxacin (2.8-fold, *p* < 0.05) (Fig. 1*E*), consistent with elevated abundances of CmeABC, CmeDEF and other efflux-associated proteins. No difference was observed for amoxicillin+clavulanic acid or erythromycin (not shown).

**Fig. 2:**
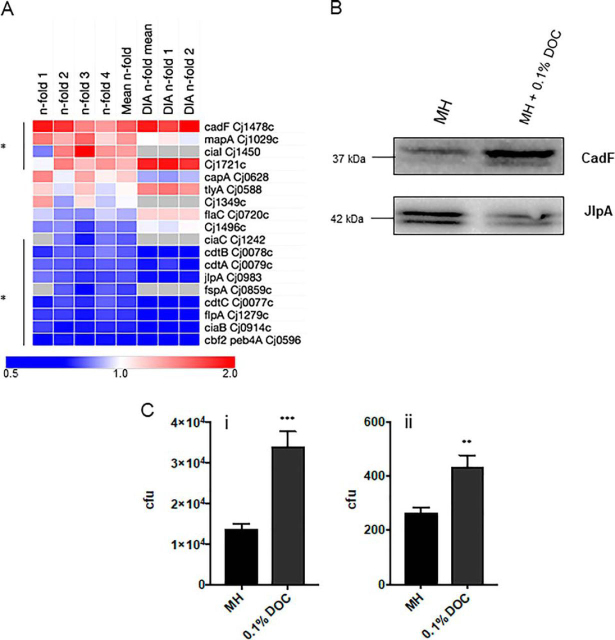
***C. jejuni* NCTC11168 growth in DOC influences abundance of virulence-associated proteins and virulence phenotypes.***A*, Heat map of known *C. jejuni* virulence-associated proteins ordered by largest mean *n*-fold change (label-based discovery; top). Data from each of 4 label-based replicates (*n*-fold 1–4) and DIA-SWATH-MS validation (“DIA” mean of 2 biological replicates [DIA *n*-fold 1–2]) are shown. Values are gray where the protein was not identified in a biological replicate and/or by DIA-SWATH MS (* denotes proteins significantly altered in abundance); *B*, Western blotting using (upper) anti-CadF and (lower) anti-JlpA serum confirming changes in their DOC abundance (JlpA appears as two major bands as it contains a single glycosylation site in NCTC11168 (addition of 1406 Da (18)) (loading controls are shown in Suppl. Fig. S3); *C*, (i) Adherence to and (ii) invasion of Caco-2 cells for *C. jejuni* passaged in 0.1% DOC (*** *p* < 0.001, ** *p* < 0.005).

Clusters associated with virulence factors were identified in both the up- and down-regulated protein data sets (Fig. 2*A*; supplemental Fig. S2A, S2B). We further validated the discovery and DIA-SWATH-MS data for CadF (1.87-fold in label-based and 2.09-fold in DIA-SWATH-MS) and JlpA (0.63-fold and 0.40-fold, respectively) by Western blotting using anti-CadF and anti-JlpA anti-sera (Fig. 2*B*). We next examined how DOC influences *C. jejuni* adherence to, and invasion of, human epithelial Caco-2 cells (Fig. 2*C*) by using gentamicin protection assays. These data show that *C. jejuni* “primed” by growth in DOC are significantly more adherent to epithelial cells and capable of increased invasion. This is consistent with previous reports (49), but also suggests some “virulence-associated” proteins may not be as crucial in this process as previously thought, whereas others (*e.g.* CadF) are validated as virulence determinants (Fig. 2*A*).

##### Changes to Nutrient Transport Proteins Reflect DOC-induced Metabolic Remodeling

**Fig. 3:**
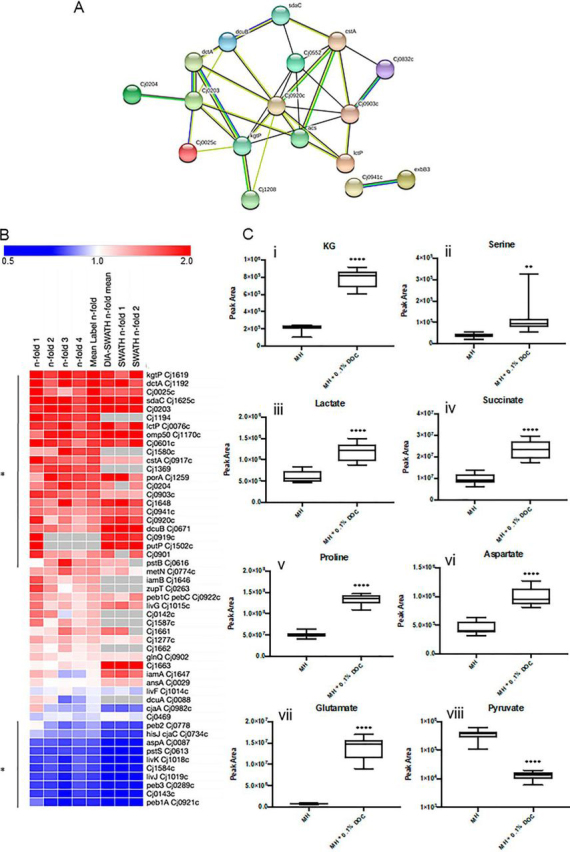
***C. jejuni* NCTC11168 growth in DOC is associated with changes in nutrient acquisition proteins that correlate with abundance of intracellular nutrients.***A*, STRINGdb cluster of nutrient transport proteins significantly elevated in abundance during growth in 0.1% DOC; *B*, Heat map of *C. jejuni* nutrient transport proteins ordered by largest mean *n*-fold change (label-based discovery; top). Data from each of 4 label-based replicates (*n*-fold 1–4) and DIA-SWATH-MS validation (“DIA” mean of 2 biological replicates [DIA *n*-fold 1–2]) are shown. Values are gray where the protein was not identified in a biological replicate and/or by DIA-SWATH MS (* denotes proteins significantly altered in abundance); *C*, (i-viii) Comparative intracellular abundances of known *C. jejuni* carbon sources following growth in MH medium with or without 0.1% DOC and measured by targeted LC-MS/MS metabolomics (**** *p* < 0.0001, ** *p* < 0.005).

STRINGdb highlighted nutrient transport as a major functional cluster associated with growth in DOC (Fig. 3*A*). Examination of the proteins contained within this cluster, the down-regulated protein set, and other characterized *C. jejuni* nutrient transporters (Fig. 3*B*) suggested that DOC induced metabolic remodeling reflected in altered abundances of specific transporters and therefore potentially uptake of their associated nutrients. LC/MS-MS targeted metabolomics (Fig. 3*C*; supplemental Data S2) detected significant increases in intracellular α-ketoglutarate (KG, transported by KgtP [2.60-fold and 2.27-fold in label-based and DIA-SWATH-MS, respectively]), Ser (SdaC [2.46-fold/2.47-fold]), Pro (PutP [2.60-fold in DIA-SWATH-MS, only identified in 1 replicate by label-based LC-MS/MS]), lactate (Lac, LctP [2.24-fold/2.52-fold]), succinate (Suc, DctA [2.57-fold/2.27-fold]/DcuB [1.69-fold/2.67-fold]/DcuA [no significant difference]) and Asp (DctA/DcuB and others). We were unable to measure citrate (Cj0203 [2.35-fold/2.40-fold]). Increased intracellular levels of alanine (Ala, hypothesized to be transported by Cj0903c [1.97-fold/1.49-fold]), Glu (associated with the Peb cluster Cj0920c-Cj0922c [Cj0920c, 1.70-fold/2.18-fold; Cj0921c, down-regulated at 0.34-fold/0.13-fold; and Cj0922c, no change in abundance), Asn and Gln were detected, whereas carbon sources with unknown transporters including pyruvate, were present at significantly decreased intracellular abundances (Fig. 3*C*, supplemental Fig. S4).

**Fig. 4:**
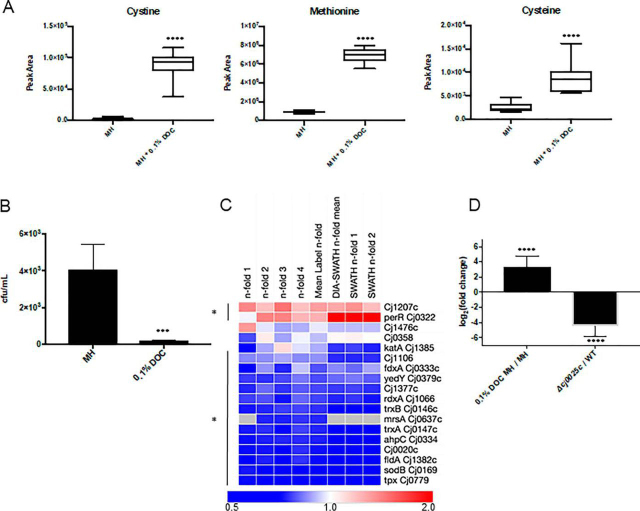
**Increased intracellular sulfur-containing amino acids in *C. jejuni* NCTC11168 grown in DOC correlate with reduced resistance to oxidative stress and increased abundance of the Cj0025c putative sodium:dicarboxylate transporter.***A*, Comparative intracellular abundances of sulfur-containing amino acids in MH medium with or without 0.1% DOC measured by targeted LC-MS/MS metabolomics (**** *p* < 0.0001); *B*, Growth in 0.1% DOC reduces resistance against oxidative stress (cells were exposed to 5 mm H_2_O_2_ for 30 min, serial dilutions were plated onto MH agar for CFU enumeration; *** *p* < 0.001); *C*, Heat map of *C. jejuni* antioxidant proteins ordered by largest mean *n*-fold change (label-based discovery; top). Data from each of 4 label-based replicates (*n*-fold 1–4) and DIA-SWATH-MS validation (“DIA” mean of 2 biological replicates [DIA *n*-fold 1–2]) are shown. Values are gray where the protein was not identified in a biological replicate and/or by DIA-SWATH MS (* denotes proteins significantly altered in abundance); *D*, qPCR showing increased *cj0025c* gene expression in 0.1% DOC and removal of *cj0025c* expression in Δ*cj0025c C. jejuni*.

Metabolomics also showed significantly increased intracellular levels of sulfur-containing compounds, including Cys, Met and the dipeptide Cys-Cys (Fig. 4*A*), as well as homoCys, biotin and thiamine (supplemental Fig. S5A–S5C). Conversely, we observed decreased acetyl-CoA, which we linked to decreased intracellular levels of both CoA and acetate, another *C. jejuni* carbon source for which no transporter is known (supplemental Fig. S5D–S5F). The organism is thought to acquire sulfur via Cys or Cys-containing dipeptides (including Cys-Gly that is transported by CptA [Cj0204; 2.02-fold/1.68-fold in 0.1% DOC]; Fig. 3*B*). Elevated intracellular sulfur is associated with susceptibility to oxidative stress (50). We observed poor survival of *C. jejuni* in the presence of 5 mm H_2_O_2_ for cells previously exposed to 0.1% DOC (Fig. 4*B*). This phenotype also correlated with reduced abundance of several antioxidant proteins, including thiol peroxidase (0.32-fold/0.17-fold), superoxide dismutase SodB (0.33-fold/0.15-fold) and alkylhydroperoxide reductase AhpC (0.44-fold/0.15-fold) (Fig. 4*C*).

##### A Putative Sodium:Dicarboxylate Transporter (Cj0025c) is Induced by DOC and Shares Sequence Similarity with the Bacterial Cystine Transporter TcyP

A significantly induced protein in 0.1% DOC was the product of the *cj0025c* gene, which is currently annotated as a “putative sodium:dicarboxylate transporter” (2.55-fold/1.97-fold; Fig. 3*B*). A BLAST search revealed Cj0025c shares 34.9% sequence identity with TcyP from *Bacillus subtilis* (supplemental Fig. S6). TcyP is a Cys-Cys transporter required for sulfur uptake (44, 51). qPCR confirmed that the increased abundance of Cj0025c was consistent with transcription of *cj0025c* during DOC growth (10.63-fold increased *cj0025c* expression; Fig. 4*D*). A deletion mutant of *cj0025c* (Δ*cj0025c*) was produced in *C. jejuni* NCTC11168 and loss of gene expression confirmed by qPCR (-22.20-fold decrease; Fig. 4*D*).

##### Δcj0025c is Attenuated for Cys-Cys Uptake Compared with Wild-type C. jejuni

**Fig. 5:**
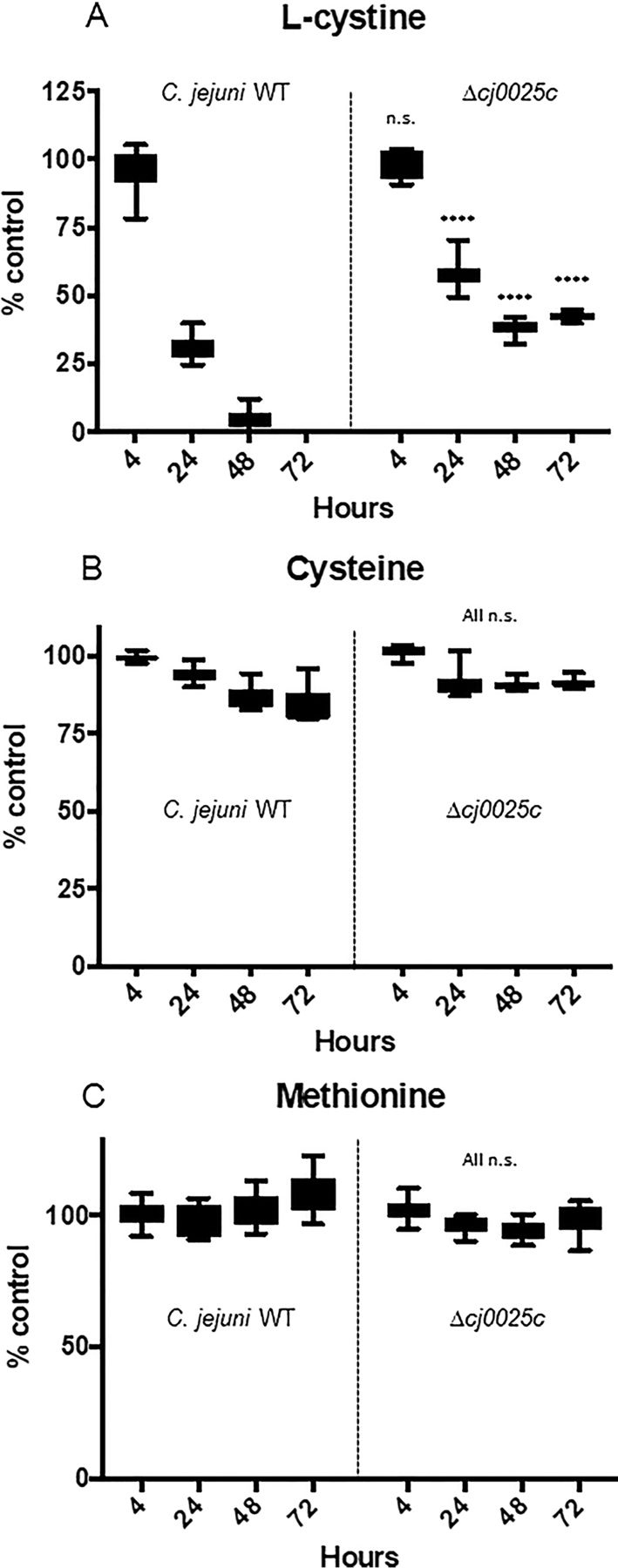
**Culture supernatant levels of sulfur-containing amino acids for *C. jejuni* NCTC11168 WT and Δ*cj0025c* grown in MCLMAN medium across 0–72 h growth**. (*A*) l-cystine; (*B*) cysteine; (C*)* methionine (**** *p* < 0.0001, n.s. not significant). Data expressed at each time point as a % of the uninoculated control (% control). Uninoculated controls were incubated identically and measurements for each amino acid taken at the same time-points, no significant changes were observed (data not shown).

Given the correlation between DOC-induced Cj0025c abundance and increased intracellular Cys-Cys, we next tested whether deletion of *cj0025c* influenced Cys-Cys uptake by monitoring culture supernatant levels in defined medium using targeted metabolomics over a time-course of *C. jejuni* growth from 0–72 h. Culture supernatant Cys-Cys levels were maintained at ∼100% of uninoculated medium in both wild-type (WT) and Δ*cj0025c* after 4 h growth. By 24 h however, medium Cys-Cys was maintained to ∼57% of the control for Δ*cj0025c*, whereas only ∼27% for WT. Furthermore, at both 48 and 72 h, Cys-Cys was almost, or completely, depleted from the medium in the WT yet maintained at ∼43–47% of the control in Δ*cj0025c* (Fig. 5*A*). Although this provides strong evidence that Cj0025c is involved in Cys-Cys uptake, we also needed to confirm that Cys-Cys was not being converted to Cys in the medium. Metabolomics confirmed that no change in medium Cys levels occurred in either WT or Δ*cj0025c* and that Cys was maintained at the levels of the uninoculated control in the presence of Cys-Cys (Fig. 5*B*). Additionally, no changes were observed for extracellular Met (Fig. 5*C*).

##### Cj0025c is Not Required for Uptake of Known C. jejuni Carbon Sources

Since there are carbon sources (*e.g.* pyruvate, malate) for which a transporter has not been identified in *C. jejuni*, we wished to determine whether Cys-Cys was the sole nutrient transported by Cj0025c. It is also possible that Cj0025c is a secondary transporter for a carbon source imported by other proteins. We measured culture supernatant levels of known carbon sources in *C. jejuni* over 0–72 h. Our data showed no difference between WT and Δ*cj0025c* uptake for Ser at every time point (supplemental Fig. S7A) and Ser was the most rapidly depleted nutrient for both strains, consistent with this amino acid being the preferred carbon source for *C. jejuni*. We observed slower uptake of Pro and Glu in Δ*cj0025c* at 24 h (and Asn at 4 h), and more rapid uptake of Asp in Δ*cj0025c* at the same time point; however by 48 h all 4 amino acids were depleted in both strains (supplemental Fig. S7B–E). We saw no uptake of Gln, which is consistent with *C. jejuni* NCTC11168 lacking a secreted GGT (supplemental Fig. S7F). Culture supernatant levels of the organic acids citrate, fumarate and KG did not differ at any time point (supplemental Fig. S8A–S8C). For pyruvate and malate, we observed slower uptake in Δ*cj0025c* at 24 h and 4 h, respectively; but each nutrient was depleted in both strains by 48 h (supplemental Fig. S8D–S8E). We also confirmed that both WT and Δ*cj0025c* underwent the “succinate shift” where Asp and fumarate are converted within the cell to succinate, which is then exported *via* DcuB into the medium, for subsequent re-uptake *via* DctA, which enables succinate use as a primary carbon source (36, 52). Medium succinate peaked at 200–300% the levels seen in uninoculated controls between 4–24 h in both strains (supplemental Fig. S8F). The rate of re-uptake was slower in Δ*cj0025c* (observed at 24 h) but succinate was depleted in both WT and Δ*cj0025c* by 48 h. Finally, we observed reduced depletion of Lac for Δ*cj0025c* compared with WT (supplemental Fig. S8G), with medium Lac maintained at ∼10–20% of the uninoculated control at 48 and 72 h.

##### The Proteome of Δcj0025c Reflects a Sulfur Starved Phenotype

**Fig. 6:**
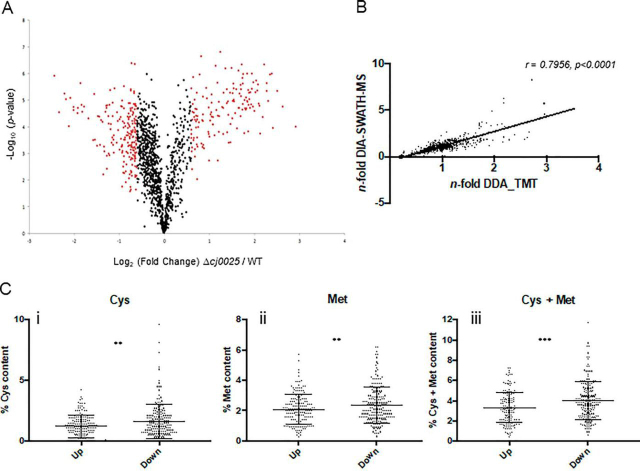
**Deletion of *cj0025c* alters the *C. jejuni* NCTC11168 proteome consistent with sulfur starvation.***A*, Volcano plot for Δ*cj0025c* compared with WT *C. jejuni*; *x* axis represents averaged Log_2_(Δ*cj0025c*/WT), *y* axis represents log_10_(*p* value). Significantly differentially abundant proteins are highlighted in red (*p* < 0.05); *B*, Correlation plot based on *n*-fold changes observed in the discovery set (*n*-fold DDA_TMT) and validation set (*n*-fold DIA-SWATH-MS), Pearson correlation *r* = 0.7956 (*p* < 0.0001) determined from 983 aligned proteins; *C*, % sulfur-containing amino acid content for proteins significantly increased (Up) and decreased (Down) in abundance in Δ*cj0025c* compared with WT; (i) cysteine, (ii) methionine, (iii) cysteine + methionine (** *p* < 0.005, *** *p* < 0.001).

We next hypothesized that if *cj0025c* encodes a TcyP-like Cys-Cys transporter, then the proteome of Δ*cj0025c* should reflect a sulfur starved phenotype and show depletion of proteins involved in Cys/Met metabolism and biosynthesis, Fe-S cluster proteins and biosynthesis of sulfur-dependent compounds, such as *S*-adenosylmethionine (SAM), molybdopterins, thiamine, biotin and CoA, as well as processes dependent on these. We conducted label-based discovery and DIA-SWATH-MS validation proteomics as described above. Label-based discovery LC-MS/MS quantified 1349 proteins (≥ 2 peptides *per* protein; 83.1% of the predicted *C. jejuni* NCTC11168 proteome) from a minimum of 2/5 biological replicates (Fig. 6*A*, supplemental Data S3). System-wide validation using additional biological replicates and DIA-SWATH-MS enabled the independent relative quantitation of 983 proteins (60.6% of the predicted proteome; supplemental Data S3). The label-based and DIA-SWATH-MS data showed a Pearson correlation of *r* = 0.7956 (Fig. 6*B*; supplemental Fig. S9). Deletion of *cj0025c* resulted in 371 proteins demonstrating a significant change in abundance (182 elevated and 189 reduced in abundance; Fig. 6*A*, supplemental Table S2). We observed large increases in abundances of the products of genes from within phase variable gene clusters for which no DIA-SWATH-MS validation was observed (supplemental Fig. S9), suggesting these are of low abundance in the wild-type. Analysis of the “changing” protein data set identified 145 proteins (39.1% of altered proteins; 75 increased in abundance and 70 decreased in abundance) that are involved in sulfur-associated or -dependent processes (supplemental Table S2). We next examined the Cys/Met content of proteins within this data set because bacterial sulfur starvation can result in changed expression of proteins containing high or low content sulfur-containing amino acids (53, 54). Proteins decreased in abundance in Δ*cj0025c* had a statistically significantly higher % Cys, % Met (both *p* < 0.01) and % Cys+Met (*p* < 0.005) content than those increased in abundance (Fig. 6*C*).

Finally, we compared the DOC and Δ*cj0025c* data sets and identified 48 proteins with significant and opposite fold changes (15 elevated in DOC and reduced in Δ*cj0025c* [possibly promoted by Cys-Cys uptake/sulfur] and 33 reduced in DOC and elevated in Δ*cj0025c* [possibly repressed by Cys-Cys uptake/sulfur]; supplemental Fig. S10); of these, 23 (47.9%) were associated with sulfur-related functions (supplemental Table S2), indicating enrichment of such proteins compared with the Δ*cj0025c* set alone. There was no association with any functional group in the Cys-Cys/sulfur ‘promoted’ set (although several proteins with large magnitude changes were sulfur-associated [*e.g.* Cj0908 is a predicted Fe-S cluster protein]), however in the Cys-Cys/sulfur ‘repressed’ set we observed a group of antioxidant proteins (Tpx, SodB, Cj0012c, FldA, TrxB and RdxA), as well as proteins involved in Fe-S cluster repair and sulfur delivery to molybdenum (NifU, IscS, FdhD, ModA, Cj1516 and Cj1517 [MoaD]). These data provide further evidence of a role for Cj0025c in Cys-Cys transport.

##### Deletion of cj0025c Disrupts Wild-type Virulence Phenotypes

**Fig. 7:**
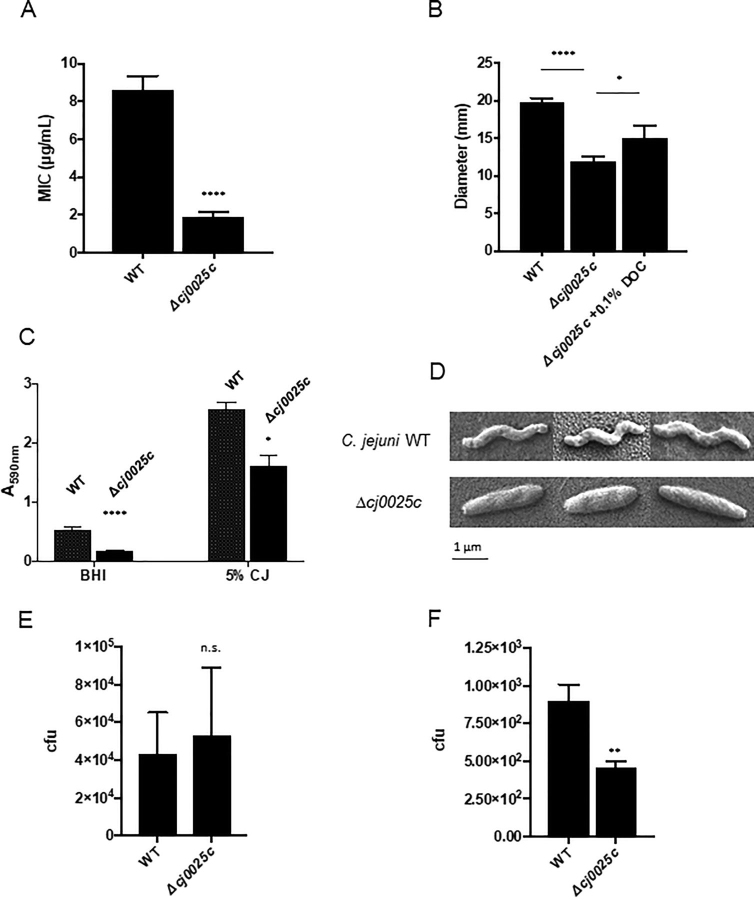
**Deletion of *cj0025c* results in altered *C. jejuni* NCTC11168 phenotypes associated with virulence.***A*, Polymyxin B resistance assay (**** *p* < 0.0001); *B*, Motility assay based on colony diameter in mm on semi-solid agar (**** *p* < 0.0001, * *p* < 0.05); *C*, Biofilm assays in (left) BHI medium and (right) BHI medium supplemented with 5% chicken exudate (‘juice’ [CJ]; **** *p* < 0.0001, * *p* < 0.05); *D*, Cell morphology assayed by S.E.; *E*, *C. jejuni* Caco-2 cell adherence and (*F*) invasion (** *p* < 0.005, n.s. not significant).

We next phenotypically characterized *C. jejuni* following deletion of *cj0025c*. Δ*cj0025c* was attenuated for polymyxin B resistance (Fig. 7*A*), consistent with significantly reduced abundances of Cme and other efflux associated proteins (supplemental Fig. S11A). Δ*cj0025c C. jejuni* also displayed significantly reduced motility (Fig. 7*B*). Addition of 0.1% DOC however, restored motility toward WT levels, which suggests Cj0025c is not required for the DOC-associated increase in motility we observed above (Fig. 1*D*). Δ*cj0025c* was unable to form biofilm at WT levels in two models of biofilm growth - one in BHI medium (3-fold reduction) and the other in medium supplemented with 5% chicken exudate (“juice” [CJ; 1.6-fold reduction in biofilm]), which increases *C. jejuni* biofilm formation (43) (Fig. 7*C*). This was consistent with our proteomics data showing significant changes to both SAM-dependent proteins and those involved in signal molecule synthesis and biofilm formation (supplemental Table S2). S.E. revealed a loss of typical *C. jejuni* helical cell shape, with Δ*cj0025c* displaying a swollen, rod-like appearance (Fig. 7*D*). Analysis of the proteomics data for shape determinants (supplemental Fig. S11B), indicated that the morphological data were consistent with a significant decrease in abundance of Cj1345c Pgp1 (0.19-fold and 0.04-fold) (55). Despite an increase in the abundance of the phosphoethanolamine (pEtN) transferase EptC that has been shown to modify lipid A, the flagellar protein FlgG and the *C. jejuni N*-linked glycan (40, 56), we observed no change in the composition or abundance of lipid A variants in Δ*cj0025c* compared with WT (supplemental Fig. S12). Given the reduction of wild-type traits associated with virulence, we tested the ability of WT and Δ*cj0025c* to adhere to, and invade, Caco-2 cells. Δ*cj0025c* showed no significant loss of Caco-2 cell adherence (Fig. 7*E*), but was attenuated for cell invasion, showing an ∼2-fold reduction in intracellular numbers as determined by gentamicin protection assay (Fig. 7*F*). These data correlated with the lack of altered protein abundance seen for CadF, but reduced abundances of the Cdt proteins in Δ*cj0025c* (supplemental Fig. S11C).

##### Addition of a Toxic Cys-Cys Mimic Inhibits Growth of WT But Not Δcj0025c C. jejuni

**Fig. 8:**
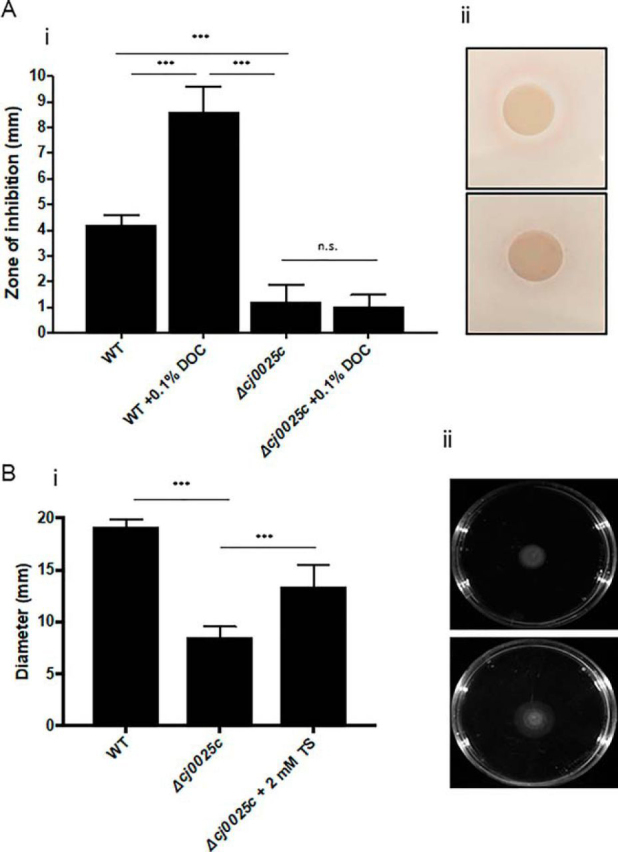
**Cj0025c is a TcyP-like Cys-Cys transport protein.***A*, Selenocystine inhibition assay (i) Se-Cys-Cys inhibits WT *C. jejuni* and 0.1% DOC increases inhibition consistent with increased abundance of Cj0025c under 0.1% DOC growth (Fig. 3*B*), whereas Δ*cj0025c* is only slightly inhibited and 0.1% DOC does not influence this phenotype (*** *p* < 0.001, n.s. not significant), (ii) representative Se-Cys-Cys inhibition plates (upper) *C. jejuni* WT and (lower) Δ*cj0025c*; *B*, Provision of an alternative sulfur source (2 mm thiosulfate [TS]) (i) restores Δ*cj0025c* motility toward WT levels, (ii) representative motility plate assay (upper) Δ*cj0025c*, (lower) Δ*cj0025c* + 2 mm TS (*** *p* < 0.001).

Our data thus far indicated that Cj0025c encodes a TcyP homolog that is able to transport Cys-Cys as a sulfur source for *C. jejuni* NCTC11168. We next employed a selenocystine (Se-Cys-Cys) inhibition assay. Se-Cys-Cys is a toxic Cys-Cys analog that inhibits bacterial growth (44) by incorporation of Se into Fe-S cluster proteins. Se-Cys-Cys inhibition plate assays of WT and Δ*cj0025c*, and in medium supplemented with 0.1% DOC, were performed with the hypothesis that DOC elevated Cj0025c protein levels (Fig. 3*B*) and thus would increase Se-Cys-Cys uptake and increase inhibition. Δ*cj0025c C. jejuni* were 3.5-fold more resistant to Se-Cys-Cys than WT (Fig. 8*A*). Addition of 0.1% DOC increased the Se-Cys-Cys zone of inhibition ∼2-fold for WT *C. jejuni*, consistent with the elevated abundance of Cj0025c in DOC growth, while having no influence on the Δ*cj0025c* deletion mutant. Although these data confirm a role for Cj0025c in Cys-Cys transport, Se-Cys-Cys did induce a small zone of inhibition for the Δ*cj0025c* mutant providing some evidence, in addition to our metabolomics assays, that a second, potentially lower affinity, Cys-Cys transporter may be present in *C. jejuni. C. jejuni* can utilize alternative sulfur sources, including hydrogen sulfide and thiosulfate. We determined whether provision of thiosulfate (TS) could functionally complement Δ*cj0025c* and addition of 2 mm TS resulted in significant recovery of motility (∼1.5-fold increase; *p* < 0.005) in Δ*cj0025c* (Fig. 8*B*), without completely restoring this phenotype to WT levels.

## DISCUSSION

*C. jejuni* encounters a hostile environment upon colonization of the human gut, facing chemical and biological factors that must be overcome to establish infection, including bile salts and the presence of established microflora, which compete for nutrients and employ toxic products to inhibit foreign microorganisms. We examined the proteome response of *C. jejuni* to the bile salt DOC. DOC induced a proteome response indicative of *C. jejuni* being primed for epithelial cell colonization that is largely in agreement with transcriptomic studies of DOC growth (49). *C. jejuni* increased abundance of a functionally undefined transporter, Cj0025c, which we have shown is required for WT uptake of Cys-Cys and for several virulence-associated phenotypes, most likely driven by a requirement for sulfur to be incorporated into small molecules that drive virulence processes, including motility and biofilm formation.

DOC positively influenced virulence traits including resistance to polymyxin B and ciprofloxacin. These data aligned with elevated abundances of proteins related to antibiotic efflux. Antibiotic resistance is largely driven by the tripartite efflux system CmeABC, which has been associated with resistance to bile salts (2 mm choleate) (57). Elevated transcription of *cmeABC* has also been observed upon bile salt exposure (49). *cmeB* negative *C. jejuni* are more susceptible to ciprofloxacin (58), which is consistent with our data showing elevated CmeABC and increased resistance to this fluoroquinolone. Despite this, *cmeB* mutants did not show any difference with respect to polymyxin B resistance suggesting DOC-associated resistance to this antimicrobial peptide may be independent of CmeABC.

*C. jejuni* exposed to DOC demonstrated significantly higher Caco-2 cell adherence and invasion. These data support the critical role of the adhesin CadF in *C. jejuni* pathogenesis (14, 15). DOC-associated increases in invasion were initially difficult to reconcile with the proteomics data, which showed no change or significantly decreased abundances of CiaBC and CdtABC. Previous work examining the transcriptomics response of *C. jejuni* to DOC indicated that exposure had no effect on *cadF* expression or adherence (to INT407 cells), but resulted in increased *ciaB* expression and invasion (49). Cia and Cdt proteins are secreted, whereas our experiments measured their intracellular levels. Protein secretion in *C. jejuni* is poorly understood but is thought to occur *via* a type III secretion system (T3SS)-like flagellar export apparatus (30, 60). Proteomics for these and other ‘secretion-associated proteins’ revealed most were elevated in DOC (supplemental Fig. S13A), which suggests increased secretion, rather than reduced expression, may be responsible for intracellular abundances of ‘invasion’ proteins. This agrees with (21) who showed increased DOC-generated secretion of CiaB.

The largest protein cluster that changed in response to DOC was nutrient transporters, suggesting bile salts induce uptake of gut-associated carbon sources and trace elements. *C. jejuni* likely adapts to the gut by elevating expression of transporters specific for mucin-associated amino acids, such as Ser and Pro, as well as organic acids produced as by-products by resident microflora [28, 29,61]. *C. jejuni* increased abundance of SdaC and PutP in DOC, resulting in increased intracellular Ser and Pro. *sdaC* deletion mutants are attenuated for chicken colonization and mouse intestinal model virulence (34, 35), whereas those defective for Pro uptake are attenuated in the mouse alone. Transporters for organic acids were also elevated in DOC, including KgtP, LctP and DctA/DcuB, and these were also correlated with increased intracellular levels of their specific nutrients. Lactate is more abundant in the upper gastrointestinal tract and may act as an environmental cue that allows *C. jejuni* to determine an optimal niche during infection (62). Conversely, we observed significantly decreased intracellular acetate and pyruvate, two carbon sources for which transporters are yet to be elucidated. *C. jejuni* also produces pyruvate from both Lac and Ser (28, 29), however increases of these did not result in elevated pyruvate. Proteins responsible for production of pyruvate from Lac and Ser were largely unchanged in DOC (data not shown), however we did observe reduced abundances of phosphoenolpyruvate carboxylase (PckA) and the Cj0073c-Cj0075c membrane lactate dehydrogenase-like complex (supplemental Data S1) (37).

DOC increased intracellular abundances of several sulfur-containing compounds, including Cys, Met and Cys-Cys, although decreased acetyl-CoA was observed. Acetate uptake is linked to acetyl-CoA production for maintenance of the TCA cycle and we observed significantly induced DOC levels of acetyl-CoA synthase (Acs; Suppl. Data S1), suggesting that lower acetate may account for intracellular acetyl-CoA. Acquisition of sulfur is crucial for colonization, with the gut rich in sulfated biomolecules including mucins, free dietary Cys and the bile component taurocholic acid (63). Gut microflora also produce hydrogen sulfide as a by-product of sulfur metabolism. *C. jejuni* is capable of utilizing 0.5–1 mm hydrogen sulfide, which is within the range encountered in the human gastrointestinal tract; however, beyond ∼2 mm growth is inhibited (38, 64). Host epithelial cells also oxidize microbiota-derived hydrogen sulfide to thiosulfate (and further oxidation in the gut to tetrathionate, although not all *C. jejuni* [*e.g.* NCTC11168] can use this sulfur source) which can also act as a sulfur source (38, 65). *C. jejuni* generally acquires sulfur from free Cys, Cys-containing dipeptides (Cys-Gly, Gly-Cys and Glu-Cys) and for some strains, GSH (29, 38). Specific transporters for most Cys-dipeptides have not been identified. In DOC, we observed elevated abundance of a putative sodium:dicarboxylate symporter, Cj0025c, which shares significant sequence similarity to the TcyP family of bacterial Cys-Cys transporters (44, 51). Deletion of *cj0025c* attenuated Cys-Cys uptake. We were surprised that low-level Cys-Cys transport was still observed in Δ*cj0025c*. This suggests the presence of a second Cys-Cys transporter, possibly the Cys-Gly transport protein CptA (Cj0204), or the *C. jejuni* carbon starvation protein A (CstA) that mediates uptake of other di- and tripeptides (66). This hypothesis was strengthened by the elevated abundance of CptA in Δ*cj0025c*. Evidence of a second Cys-Cys transporter was also demonstrated by Se-Cys-Cys assay where, despite significant protection, a small zone of inhibition remained for Δ*cj0025c*. In addition to TcyP, *E. coli* and *B. subtilis* contain ATP-binding cassette (ABC) transporters (TcyJKLMN and TcyABC in *B. subtilis* and TcyJLN in *E. coli*; (44, 50)) with affinity for Cys-Cys and other Cys-containing compounds. Unlike TcyP, we saw no similar proteins *via* BLAST search to any of these Tcy ABC transport proteins in *C. jejuni* (not shown), suggesting Cys-Cys uptake in the absence of *cj0025c* occurs *via* an unidentified process.

The Δ*cj0025c* proteome displayed changes associated with sulfur-dependent processes including redox homeostasis, Fe-S clusters, Met and SAM biosynthesis and SAM-dependent proteins, and biosynthesis of sulfur compounds (*e.g.* CoA, biotin, thiamine, molybdopterins and lipoic acid). *C. jejuni* (like other bacteria) also responded to sulfur starvation by reducing expression of high %Cys/Met-containing proteins and increasing expression of low %Cys/Met-containing proteins. Some organisms contain large gene clusters encoding proteins with low Cys/Met content (‘sulfur clusters‘), although *C. jejuni* does not contain such regions (54). Phenotypic characterization of Δ*cj0025c* indicated the attenuation of WT traits. Biofilm formation was reduced in Δ*cj0025c* consistent with a need for sulfur in SAM biosynthesis, the first step in the pathway leading to autoinducer-2 (AI-2) production for quorum sensing. Δ*luxS C. jejuni* (LuxS catalyzes the final step in AI-2 biosynthesis) are defective for motility at 37 °C (although unaffected at 42 °C), and similar AI-2 mutants in *Helicobacter pylori* are both defective for motility and show altered expression of flagellar genes (67, 68). Biofilm formation and motility are linked in *C. jejuni* (69), which may explain the similarly attenuated phenotypes observed here. Motility is a fundamental virulence determinant in *C. jejuni* and our data show that *cj0025c* is required for WT motility irrespective of LuxS, which is maintained at WT levels in Δ*cj0025c* (supplemental Data S3). Little is known regarding a link between sulfur and motility in *C. jejuni*; however, recent work in the Gram-negative pathogen *Serratia marcescens* (70) has shown that Cys, and thus sulfur, limitation reduces flagellar motility and secretion of phospholipase through the T3SS-like flagellar export apparatus, phenotypes that are correlated with sulfur-directed gene expression. We noted significantly reduced abundances of several proteins associated with flagellar export (supplemental Fig. S13B). Sulfur was also required for optimal cytotoxicity in *S. marcescens* - a phenotype we observed in Δ*cj0025c*, which was attenuated for Caco-2 cell invasion. Finally, the association of *C. jejuni* motility with sulfur could be demonstrated by supplementation with 2 mm TS, which rescued Δ*cj0025c* motility.

Helical cell shape is considered a major virulence factor. Two peptidoglycan modifying peptidases, Pgp1 (Cj1345c) and Pgp2 (Cj0906c), are responsible for cell morphology, with *pgp1* and *pgp2* negative *C. jejuni* displaying a rod-like appearance (55, 71). Transposon screens for cell shape defective *C. jejuni* have shown that single nucleotide polymorphisms or insertions/deletions within these 2 genes account for near all rod-like mutants (72, 73). An additional gene associated with curvature (Cjj81176_1105; Cj1087c in NCTC11168) and homologous to the cell shape determinant 1 (Csd1) from *H. pylori* has also been identified (74). Δ*cj0025c* showed no change to the abundance of Pgp2, whereas Cj1087c was induced and Pgp1 reduced in abundance (supplemental Fig. S11B). Increased abundance of pEtN transferase EptC was observed but could not be linked to any observable change in lipid A. We cannot unequivocally rule out the presence of a mutation in Pgp1, however our proteomics data were based on quantitation of 17 peptides (11 for DIA-SWATH-MS) spanning the full length of the predicted 52 kDa protein (data not shown), suggesting that truncation or sequence modification are not responsible for the phenotype we observed here. Peptidoglycan formation requires biosynthesis of UDP-*N*-acetylglucosamine (UDP-Glc*N*Ac) and UDP-*N*-acetylmuramic acid (UDP-Mur*N*Ac), both of which receive their acetyl group from the sulfur compound acetyl-CoA; and peptidoglycan can also be modified by *O*-acetylation mediated by PatAB (75). In DOC-treated *C. jejuni*, we saw no evidence of a change in cell morphology (not shown), however we did observe significant increases in Pgp2 and PatB abundances, which may help explain why acetyl-CoA was reduced in abundance under these conditions. Therefore, limited acetyl-CoA in sulfur starved Δ*cj0025c C. jejuni* may result in reduced availability of peptidoglycan precursors and/or an inability to acetylate mature peptidoglycan resulting in changes to cell morphology associated with Cj1087c and Pgp1.

We were surprised that no Cys uptake was observed in the presence of Cys-Cys. Several Cys transporters have been considered including CjaA and the Paq ABC transport system (Cj0467–0469) (76), however mutants in *cjaA* and *paqP* maintained WT growth in the presence of Cys as well as Cys-containing dipeptides (76, 77). Other potential Cys transporters including Peb1A (Cj0921c) and CjaC (Cj0734c) have also been reported to not transport Cys or Cys-Cys. Our data correlate with these observations because we saw no change in abundance of CjaA, CjaC or Cj0469 in DOC (where intracellular Cys and Cys-Cys are strongly increased) or Δ*cj0025c*. Additionally, Peb1A was repressed in DOC, which was also in contrast with elevated intracellular levels of Peb1A substrates, Asp and Glu, however these are transported by other proteins (*e.g.* DctA) that were induced under the same conditions. The extracellular redox balance is generally in favor of Cys-Cys (78) and we saw no evidence of inter-conversion as Cys-Cys was depleted from the medium without commensurate change in Cys. Cys-Cys drives oxidative stress in *E. coli* (50) because uptake results in rapid intracellular conversion to Cys that drives the Fenton reaction. Furthermore, over-accumulation of Cys is avoided by secretion of excess Cys into the medium. It is therefore possible that in the presence of Cj0025c, medium Cys levels are maintained because of both the intracellular conversion of Cys-Cys to Cys, release of excess Cys and concurrent Cys uptake resulting in an overall net appearance of extracellular maintenance. Given however, that we observed the same maintenance of medium Cys in Δ*cj0025c*, it appears more likely that Cys import did not occur under these conditions.

It remains possible that nutrients other than Cys-Cys may be imported *via* Cj0025c. We observed little difference between WT and Δ*cj0025c C. jejuni* for uptake of any of the amino and organic acids tested; although we did observe differences in the rate of uptake for some, and low level preservation of medium Lac in Δ*cj0025c*. Unfortunately, we did not observe the Lac permease LctP in Δ*cj0025c*, therefore we are unable to say whether this difference is a result of effects on that protein or are specific to Cj0025c. Additionally, we were unable to monitor acetate in culture medium and can make no assertions about any role for Cj0025c in acetate transport. Finally, we are also unable to conclude whether Cys-Cys is the sole dipeptide transported by Cj0025c. Given increased abundance of Cj0204/CptA upon *cj0025c* deletion, it remains plausible that there is some functional redundancy between these two transporters.

DOC induces an *in vivo*-like response at the proteome level that is reflected in increased virulence phenotypes, including metabolic rearrangement resulting in increased uptake of specific amino and organic acids, as well as sulfur compounds, including Cys-Cys. Cj0025c was induced by DOC and shares sequence identity with bacterial TcyP Cys-Cys transporters. Deletion of *cj0025c* resulted in *C. jejuni* that were attenuated for Cys-Cys import, and that display a proteome switch to a sulfur starvation-like response. Δ*cj0025c C. jejuni* were attenuated for virulence phenotypes including motility, cell morphology, antibiotic resistance, and human epithelial cell invasion, consistent with a requirement for sulfur. Resistance to the toxic analog Se-Cys-Cys confirmed that Cj0025c encodes a Cys-Cys transporter that we have named TcyP to be consistent with the nomenclature of homologous proteins in other bacteria.

## DATA AVAILABILITY

Mass spectrometry data have been deposited to the ProteomeXchange Consortium via PRIDE with the data set identifier PXD017934.
