# Supplementary material for: Proteomics of Campylobacter jejuni Growth in Deoxycholate Reveals Cj0025c as a Cystine Transport Protein Required for Wild-type Human Infection Phenotypes
Source: Mol Cell Proteomics. 2020 Nov 23;19(8):1263–80. doi: 10.1074/mcp.RA120.002029 (PMC8015009; doi:10.1074/mcp.RA120.002029)
Supplement: Supplementary file 1 [file mmc1.zip › 159411_1_supp_522819_q9nf8k.pdf]

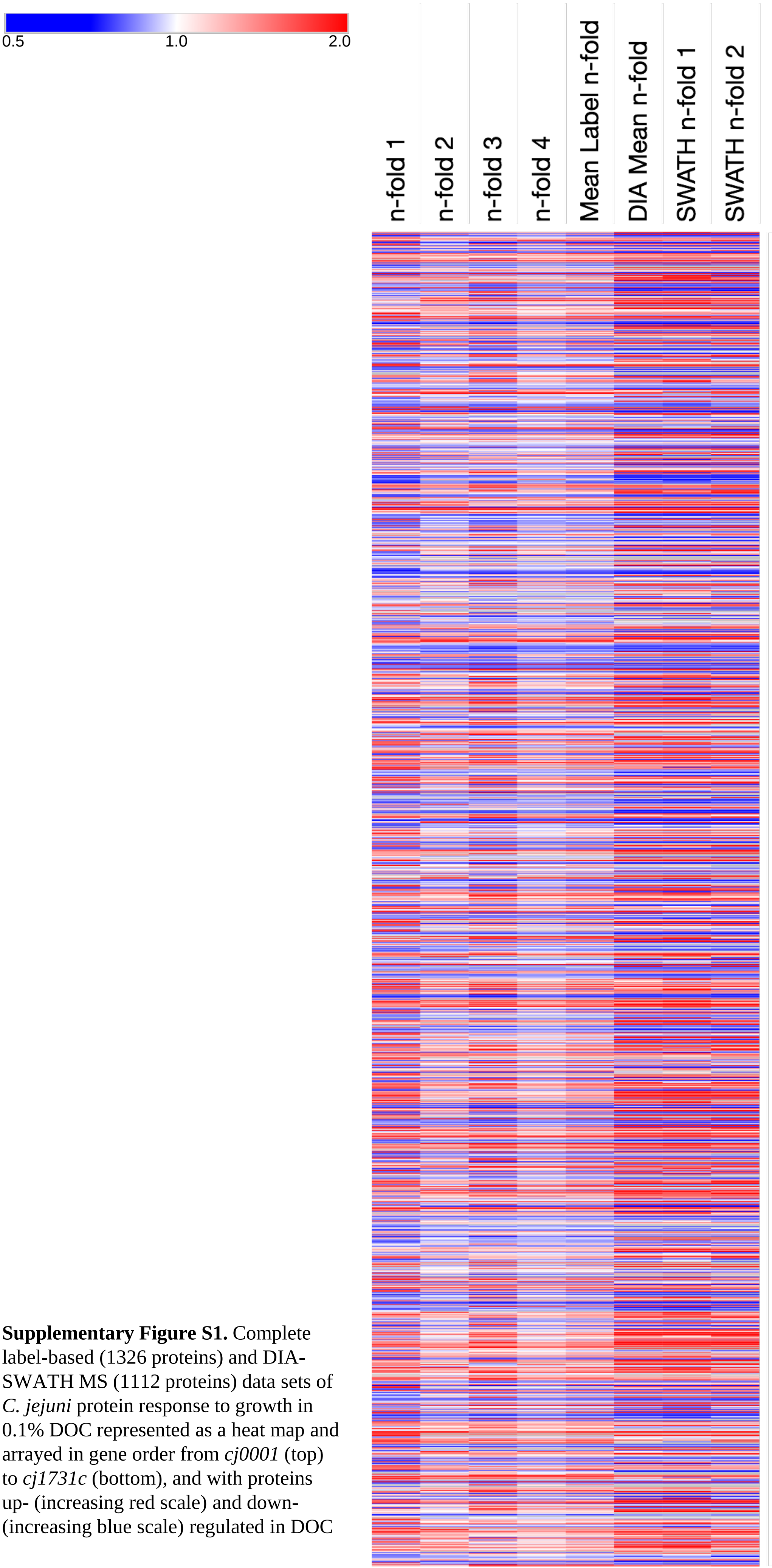

Supplementary Figure S1. Complete label-based (1326 proteins) and DIA-SWATH MS (1112 proteins) data sets of *C. jejuni* protein response to growth in 0.1% DOC represented as a heat map and arrayed in gene order from *cj0001* (top) to *cj1731c* (bottom), and with proteins up- (increasing red scale) and down- (increasing blue scale) regulated in DOC

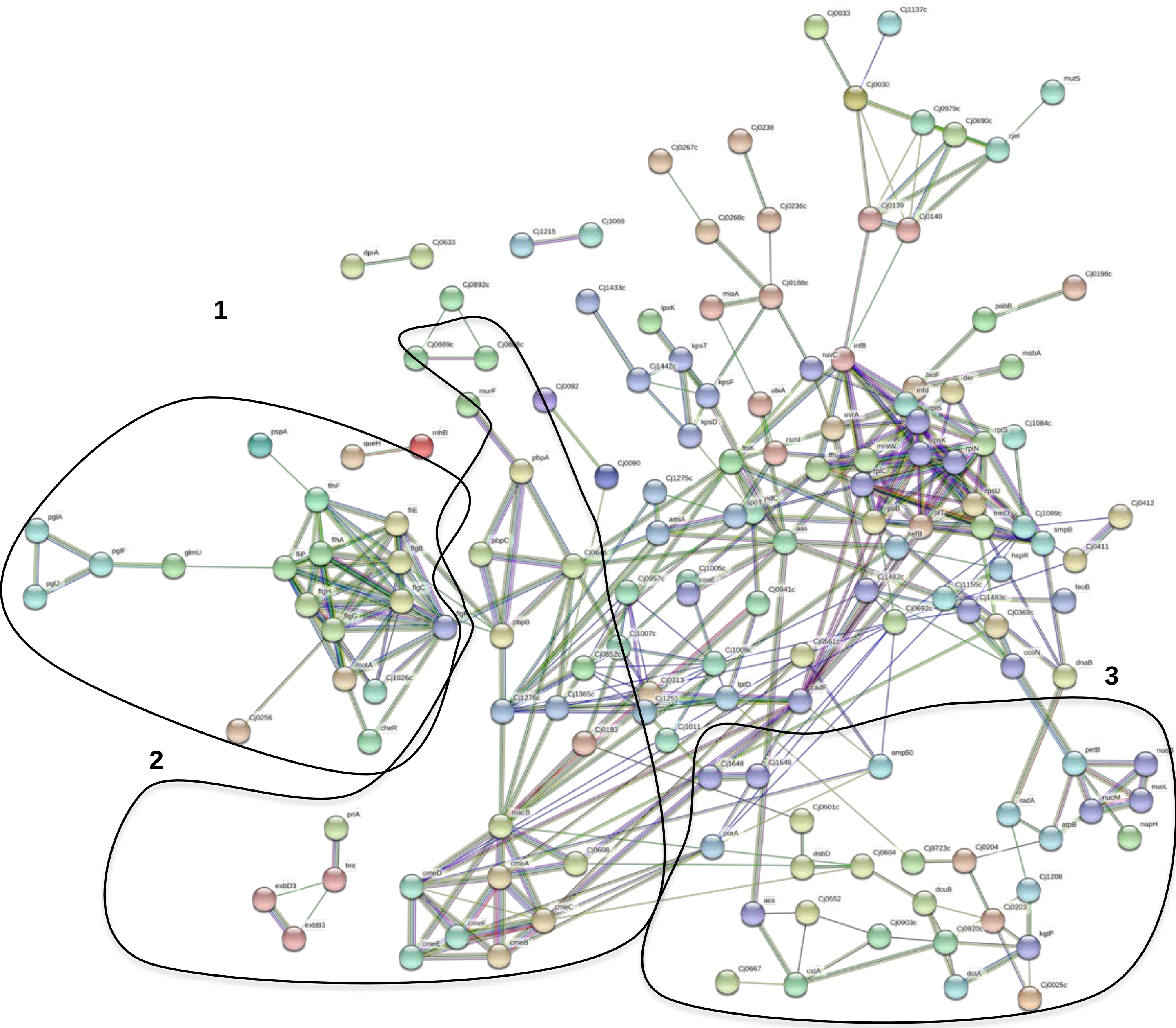

Supplementary Figure S2A. STRING db analysis of functional relationships between 244 proteins present at significantly increased abundance in *C. jejuni* NCTC11168 grown in the presence of 0.1% DOC. Major clusters were associated with motility (1), antibiotic resistance (2) and nutrient transport and utilization (3). STRING output: 244 protein nodes, 374 edges with avg. local cluster coefficient 0.471; protein-protein interaction (PPI) enrichment = 7.44e-06 at minimum required interaction score of 0.6.

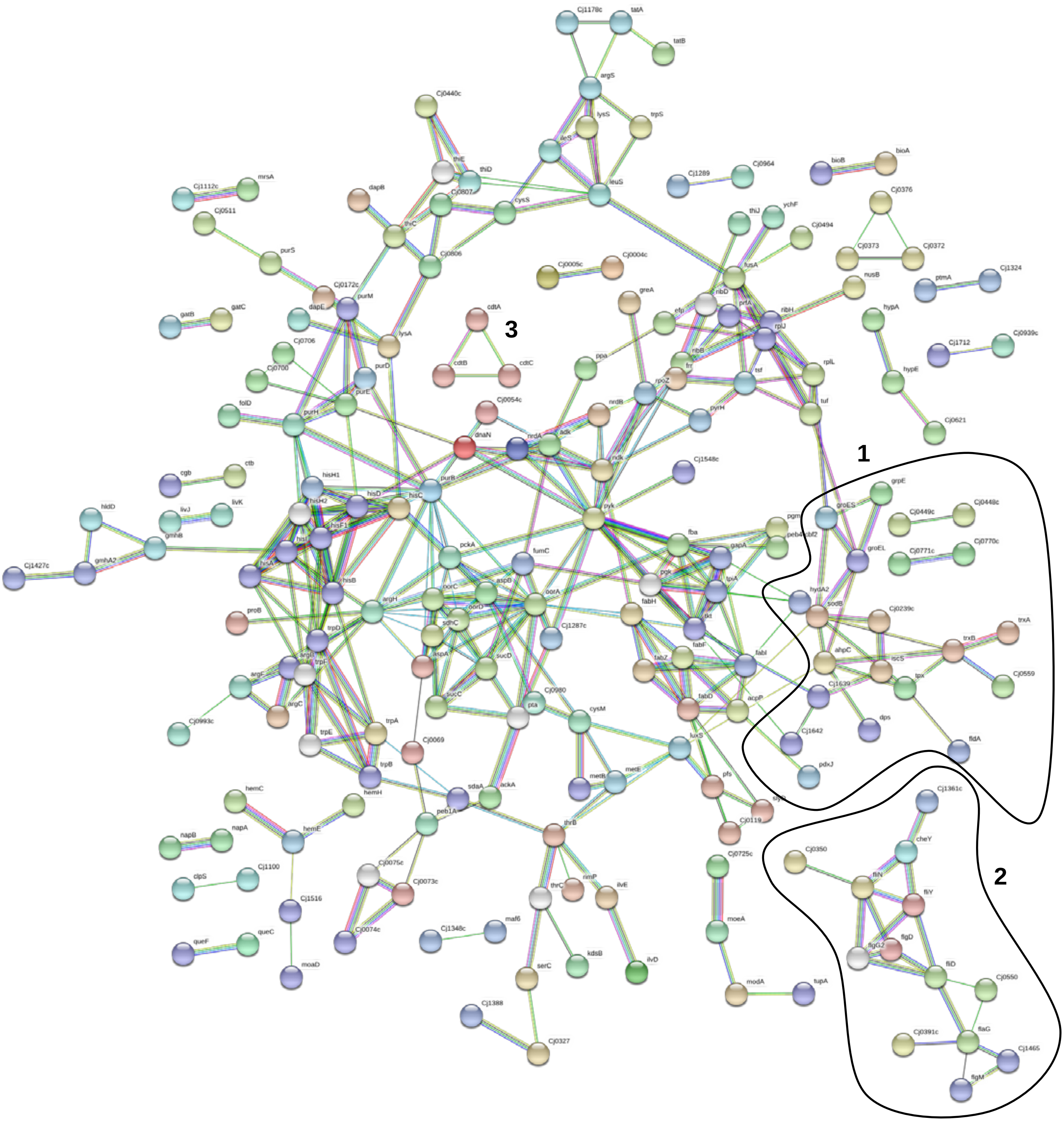

Supplementary Figure S2B. STRING db analysis of functional relationships between 268 proteins present at significantly decreased abundance in *C. jejuni* NCTC 11168 grown in the presence of 0.1% DOC. Major clusters were associated with metabolism antioxidants and stress response (1), motility (2) and virulence (e.g. Cdt proteins; 3). STRING output: 268 protein nodes, 374 edges with avg. local cluster coefficient 0.476; protein-protein interaction (PPI) enrichment = 6.90e-09 at minimum required interaction score of 0.7.

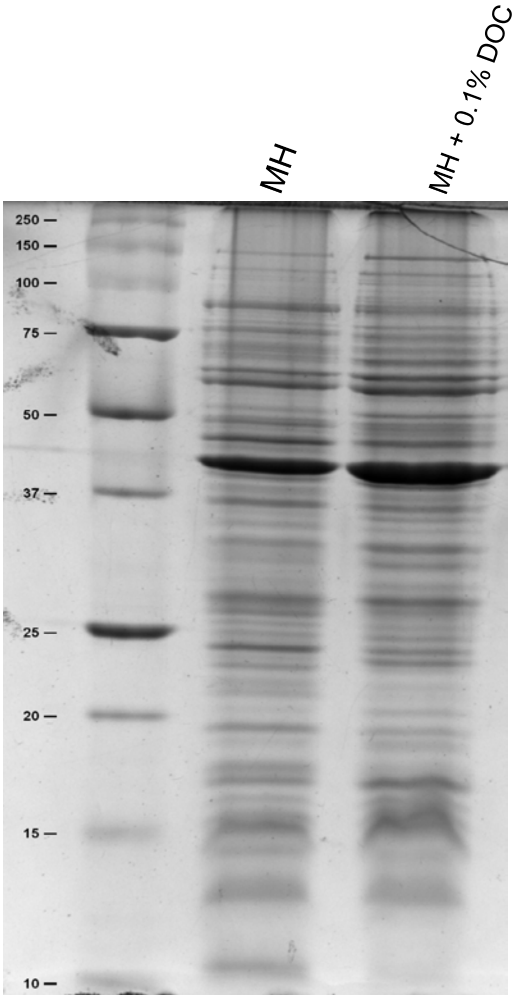

Supplementary Figure S3. Loading control for Western blots of CadF and JlpA shown in Fig. 2B. SDS-PAGE gels were stained with colloidal Coomassie Blue G-250. Left lane: molecular mass markers (Bio-Rad); middle lane: MH medium control; right lane: MH + 0.1% DOC. The band at ~40kDa represents the major outer membrane protein (PorA), which was detected as significantly up-regulated by both label-based (2.04-fold) and DIA-SWATH-MS validation (2.44-fold) approaches. Total lane densitometry for MH and MH + 0.1% DOC were within 5% of each other over *n*=3 replicates.

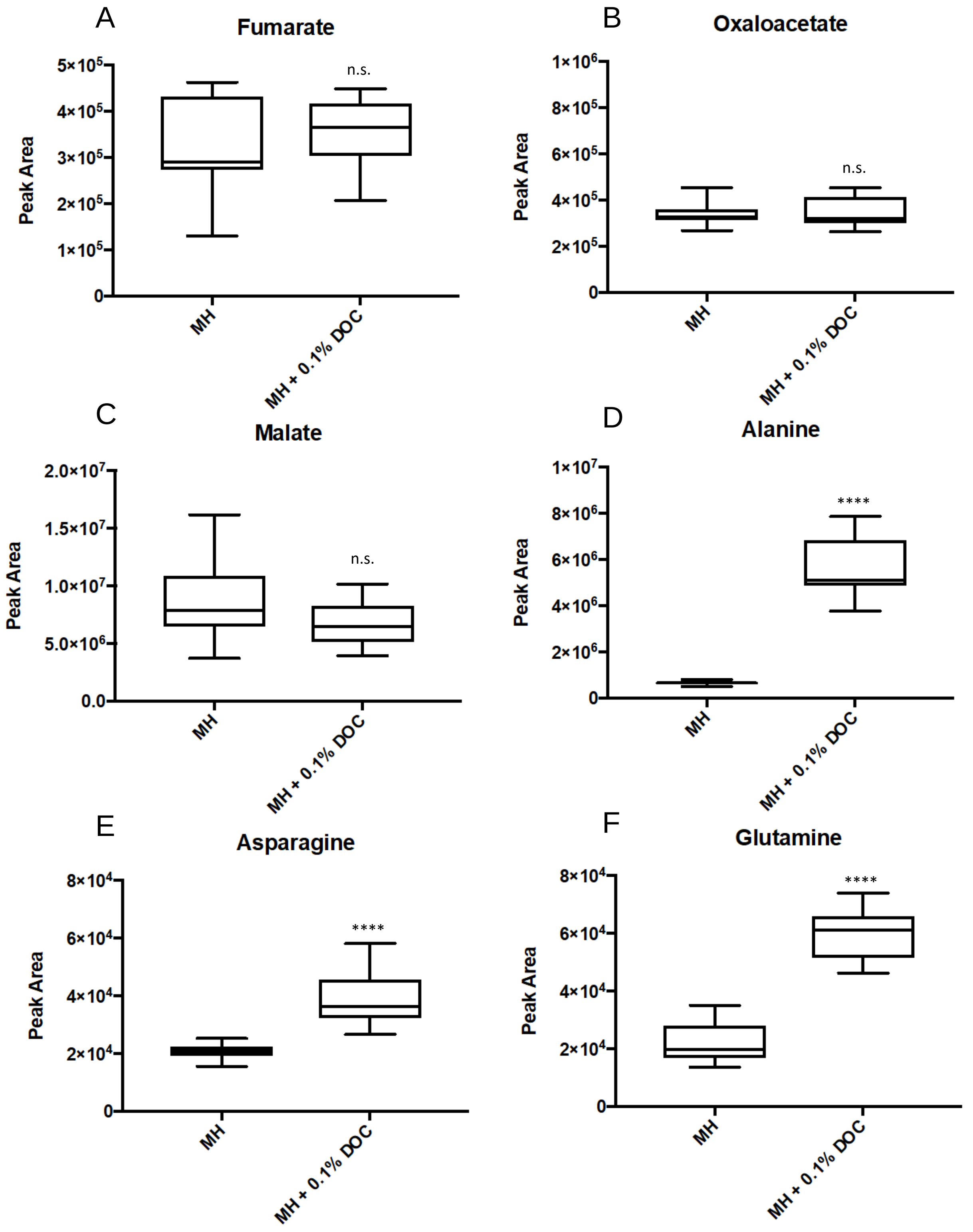

Supplementary Figure S4. Metabolomics assays of organic (A-C) and amino (D-F) acids performed by targeted LC-MS/MS from *C. jejuni* NCTC11168 in control and 0.1% DOC supplemented medium. Box and whisker plots showing peak area. **** Significantly differentially abundant metabolites (p<0.0001, fold change >+/-1.8), n.s., not significant.

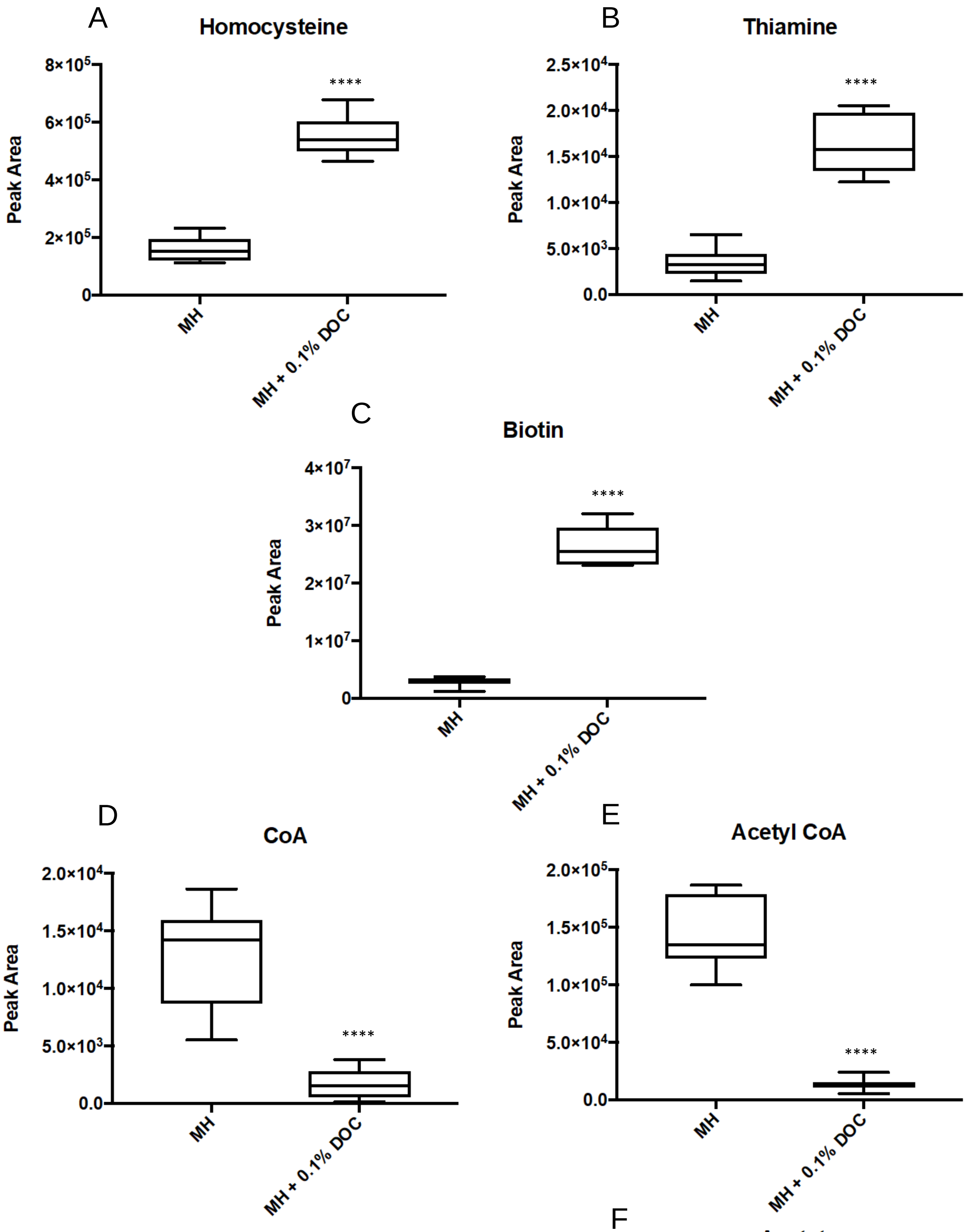

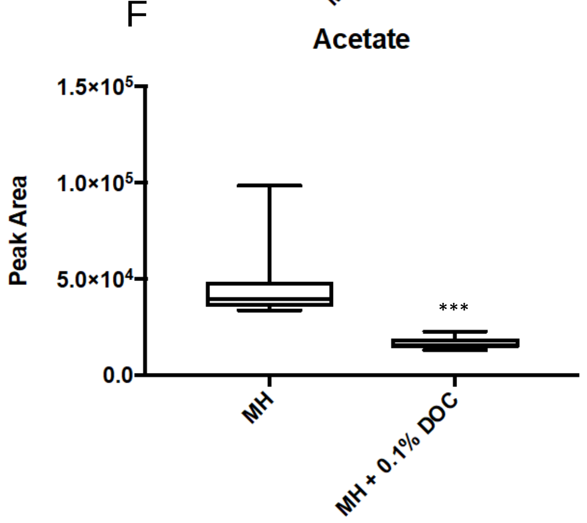

Supplementary Figure S5. Metabolomics assays of sulfur-containing compounds (A-E) and acetate (F) performed by targeted LC-MS/MS from *C. jejuni* NCTC11168 in control and 0.1% DOC supplemented medium. Box and whisker plots showing peak area. ****Significantly differentially abundant metabolites ($p<0.0001$, fold change >+/-1.8), *** ($p<0.005$), n.s., not significant.

```
TR|Q0PCA0|Cj0025c       MDKQFFQDFLMLSQAHTIATLAILCVVFYALKKMRDIKINFSLRMLFALLMGLGFGFALQ 60
TR|A0A080UK10|TcyP      -----------MATLLVILNLFIIILLIAGLFVMQKKHVSFSKRVFTALGLGIVFGFALQ 49
                                   ::   .* .* *: ::: .*  *:. ::.** *:: ** :*: ******

TR|Q0PCA0|Cj0025c       YLANFPDAEEASNILWYSETKHWFAFVSSVFVAFIKMLVVPLVSICIIKVIIEIDKNIKI 120
TR|A0A080UK10|TcyP      LIYGPASD-------IVTQTADWFNIAGGGYVKLLQMIVMPLVFISILGAFTKLKLTKNL 102
                         : .  .          ::* .** :... :* :::*:*:*** *.*: .: ::. . ::

TR|Q0PCA0|Cj0025c       SSLLGISLFWILFSTAIAATLGIFLGYSFDLGSNFAIYEGDKQIREI------------Q 168
TR|A0A080UK10|TcyP      GKISGLILGILIATTAIAAAVGIVSALSFDLQTI-QIDQGDTELSRGQELEQKSEDMTAK 161
                        ..: *: *  :: :*****::**. . **** :   * :**.:: .             :

TR|Q0PCA0|Cj0025c       TFSNIILGLIPSNIIT---AINKENIIAIVIFSFFIGISAKKISKKEEYEQAFKSFHNFI 225
TR|A0A080UK10|TcyP      TLPQQMIELLPSNPFLDFTGARPTSTIAVVIFAAFLGVAFLGVKRKQ--PEQAETFKKMV 219
                        *: : :: *:*** :    . .  . **:***: *:*::   :.:*:   :  ::*::::

TR|Q0PCA0|Cj0025c       LTFYNIMMNMTATVIRFMPYAVVCMMANVLLSNGFEAIKTAGLFIMLIYIAMFIMFGVHF 285
TR|A0A080UK10|TcyP      DAVYSIIMRVVTLILRLTPYGVLAIMTKTIATSDIDSILKLGMFVIASYAALIVMFIIHL 279
                         :.*.*:*.:.: ::*: **.*:.:*::.: :..:::* . *:*::  * *:::** :*:

TR|Q0PCA0|Cj0025c       LLLASQGLNPIKYAKKAFPVWLFAFSSRSSLGTLPMTTSTLQNKFGVNSAIANFVASIGT 345
TR|A0A080UK10|TcyP      LLVTFSGLNPFMYVKKALPVMIFAFTSRSSAGSLPLNIKT-QRSMGVPEGIANFAGSFGL 338
                        **:: .****: *.***:** :***:**** *:**:. .* *..:** ..****..*:*

TR|Q0PCA0|Cj0025c       TTGLNGCAGYFPALAAVFVAFA-THTHIDFTFALMIVLVAVIGSLGIAGVPGSATMAASI 404
TR|A0A080UK10|TcyP      SIGQNGCAGIYPAMLAIMIAPTVGQNPLDPAFIVSVIAVVAISSFGVAGVGGGATFAALL 398
                        : * ***** :**: *:::* :  :. :* :* : :: *..*.*:*:*** *.**:** :

TR|Q0PCA0|Cj0025c       MLAGIGFGNNFVMLSLILAIDPIIDMARTASNVSGAMTSALCTAKNLKALDKEIYNS--- 461
TR|A0A080UK10|TcyP      VLSALNMP--VALAGLLISVEPLIDMGRTALNVSGSMTAGLITSKATKDIDTAVFNDQST 456
                        :*:.:.:   ..: .*:::::*:***.*** ****:**:.* *:*  * :*. ::*.

TR|Q0PCA0|Cj0025c       --------
TR|A0A080UK10|TcyP      VIEAEEGA 464
```

Supplementary Figure S6. BLAST alignment of *C. jejuni* NCTC11168 Cj0025c with *B. subtilis* TcyP showing 34.9% sequence identity (* identical amino acids, : similar amino acids)

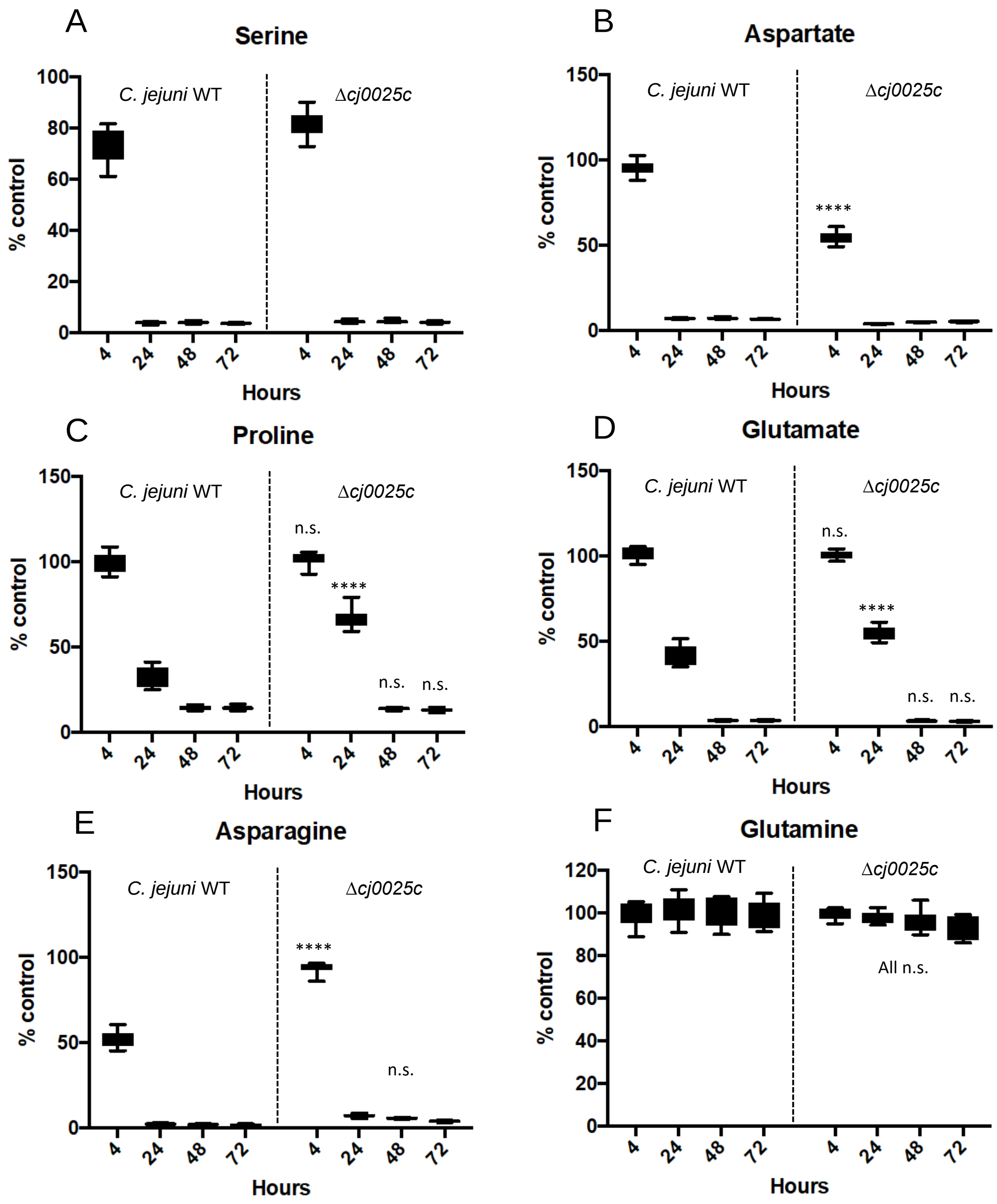

Supplementary Figure S7. Metabolomics assays of culture supernatant levels of amino acid carbon sources used by *C. jejuni* WT *and* Δ*cj0025c* performed by targeted LC-MS/MS. Box and whisker plots showing peak area. ****Significantly differentially abundant metabolites (*p*<0.0001), n.s., not significant.

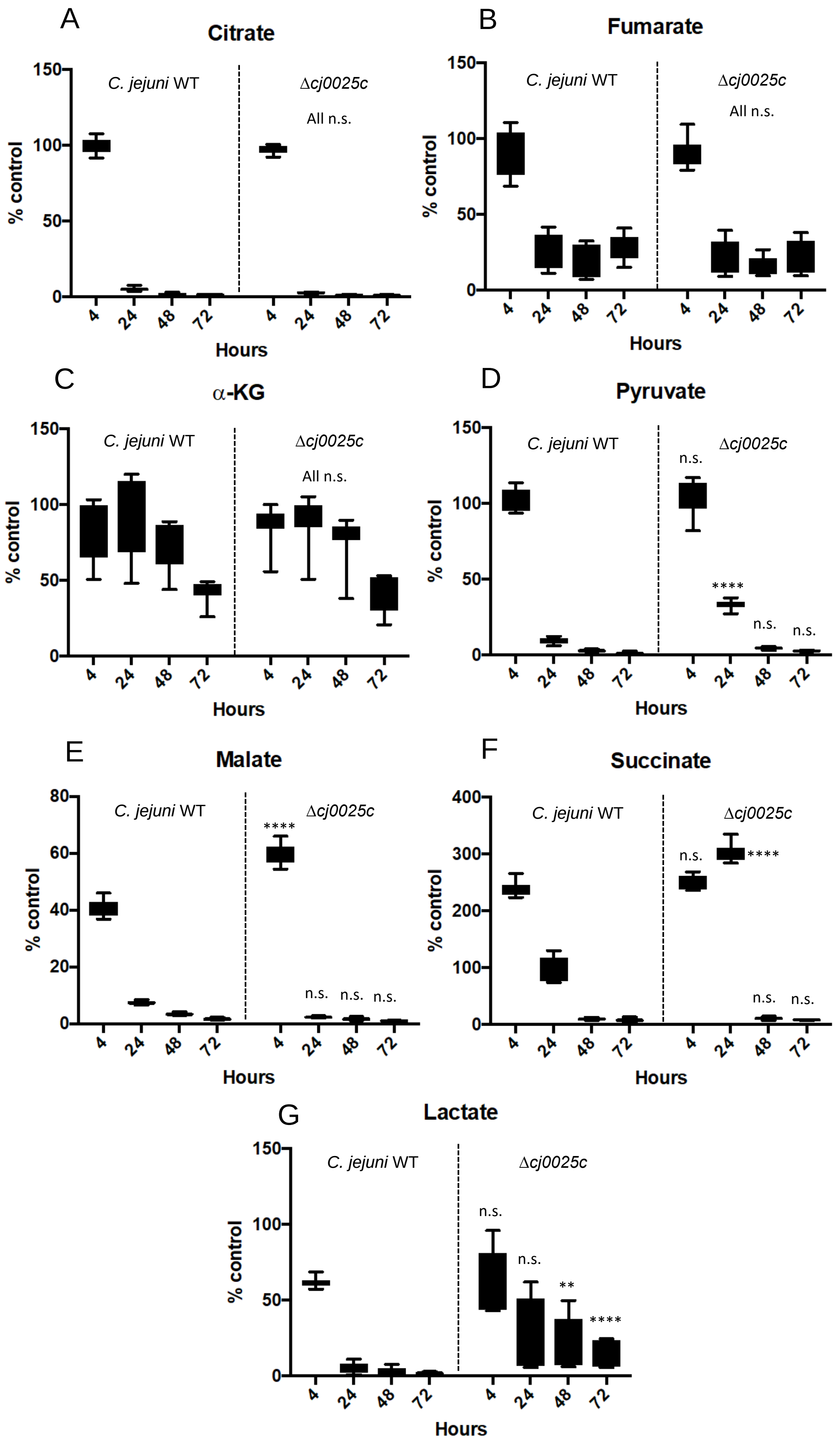

Supplementary Figure S8. Metabolomics assays of culture supernatant levels of organic acid carbon sources used by *C. jejuni* WT *and* Δ*cj0025c* performed by targeted LC-MS/MS. Box and whisker plots showing peak area. ****Significantly differentially abundant metabolites (p<0.0001), ** (p<0.001), n.s., not significant.

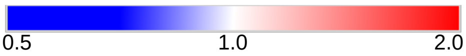

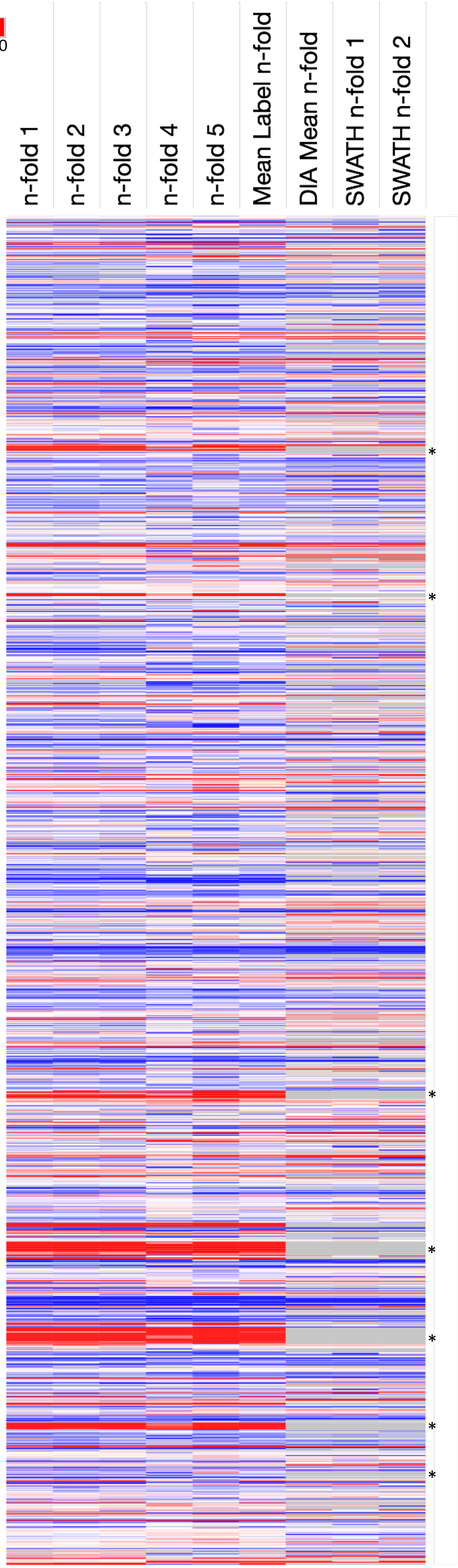

Supplementary Figure S9. Complete label-based (1349 proteins) and DIA-SWATH-MS (983 proteins) data sets of *C. jejuni* NCTC 11168 Δ*cj0025c* compared with wild-type represented as a heat map and arrayed in gene order from *cj0001* (top) to *cj1731c* (bottom). * phase variable regions identified by label-based proteomics but not in the validation cohort.

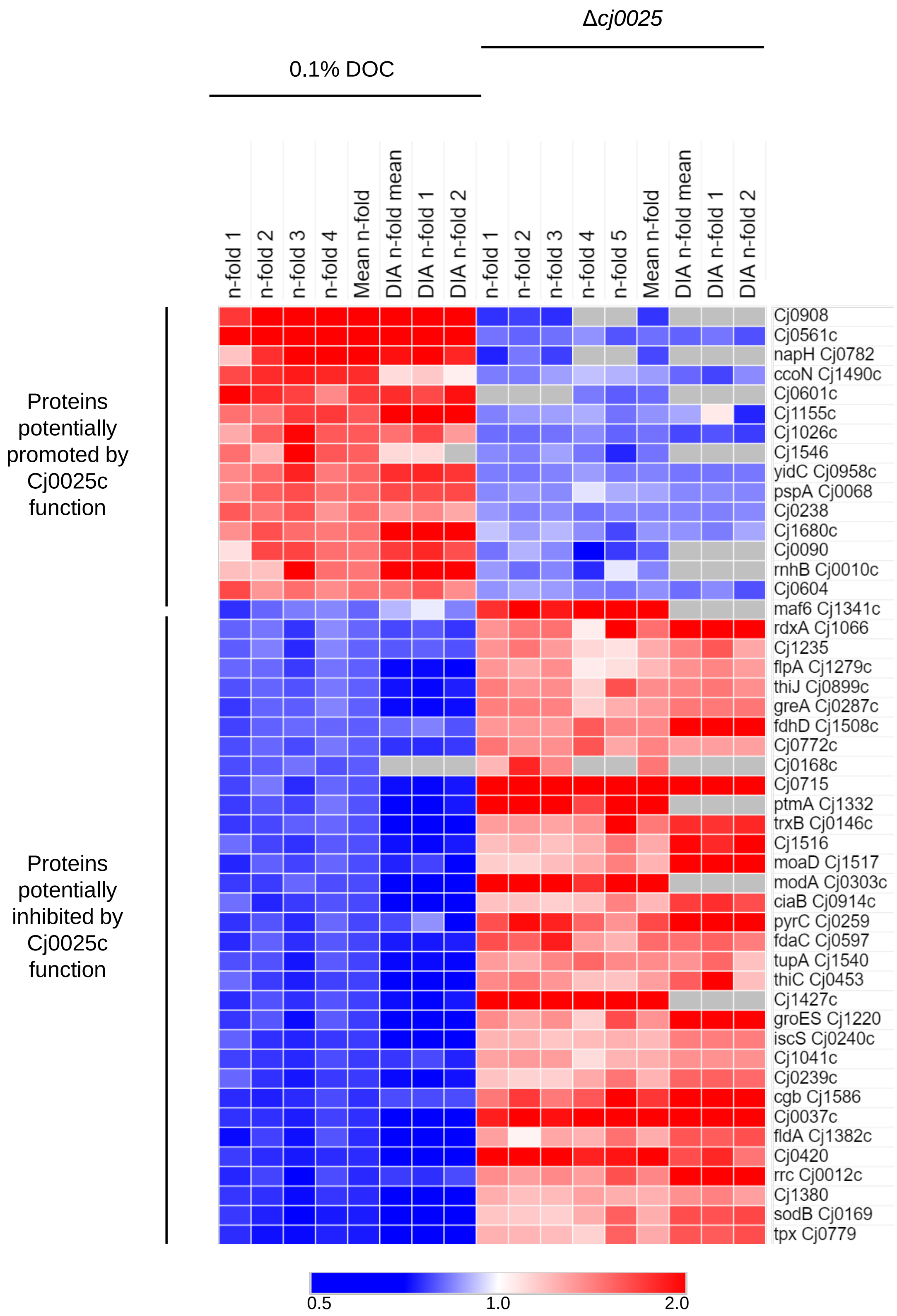

Supplementary Figure S10. Comparative heat map of proteins from quantitative proteomics data sets showing growth in 0.1% DOC versus control (0.1% DOC) and Δ*cj0025c* versus WT (Δ*cj0025c*) data sets. *n*=4 biological replicates were performed for 0.1% DOC and n=5 biological replicates were performed for Δ*cj0025c* label-based LC-MS/MS discovery (columns *n*-fold 1-4 (left); columns *n*-fold 1-5 (right), Mean *n*-fold) and a further 2 biological replicates were used for each validation by DIA-SWATH-MS (columns DIA *n*-fold mean and *n*-fold 1-2). Proteins were included based on a significant abundance change in 0.1% DOC that was reversed to an opposite (up-down or down-up) significant abundance change in Δ*cj0025c*.

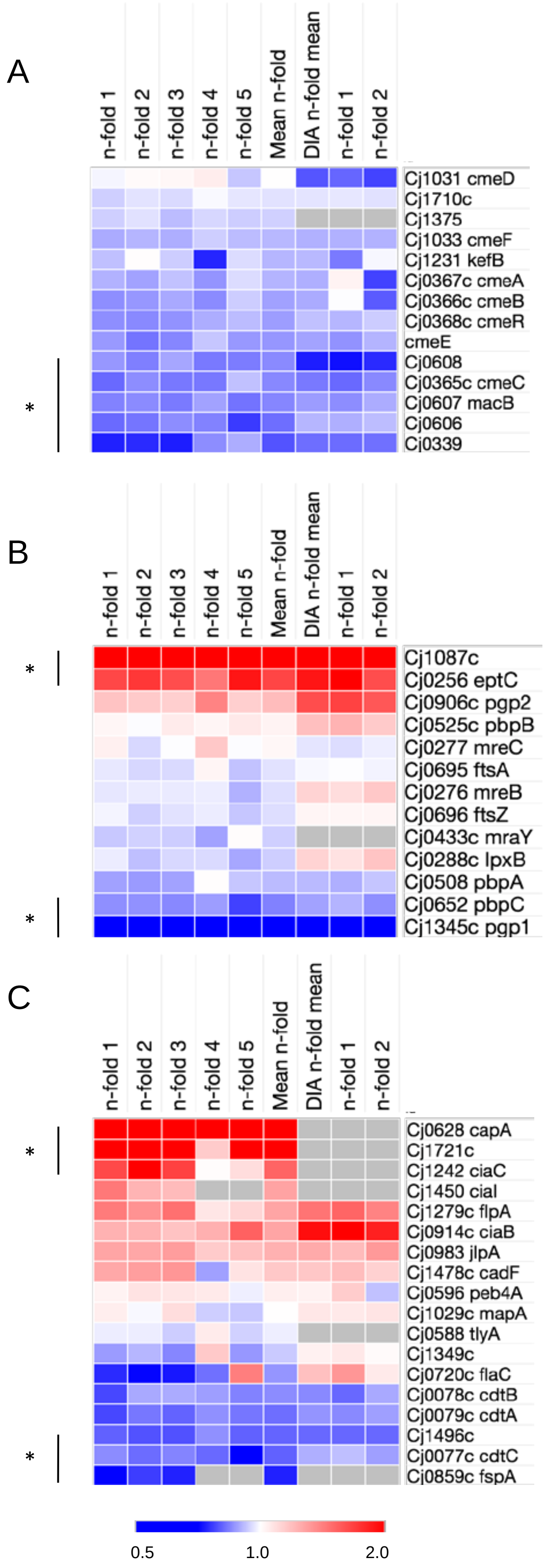

Supplementary Figure S11. Heat maps of proteins from a quantitative proteomics comparison of WT and Δ*cj0025c* *C. jejuni* NCTC11168. (A) Proteins involved in antibiotic resistance and efflux; (B) cell shape determinants; and (C) putative virulence factors. *n*=5 biological replicates were performed for label-based LC-MS/MS discovery (columns *n*-fold 1-5 (left), Mean *n*-fold) and a further 2 biological replicates were used for validation by DIA-SWATH-MS (columns DIA *n*-fold mean and *n*-fold 1-2 (right)). * Proteins deemed significantly changing in abundance.

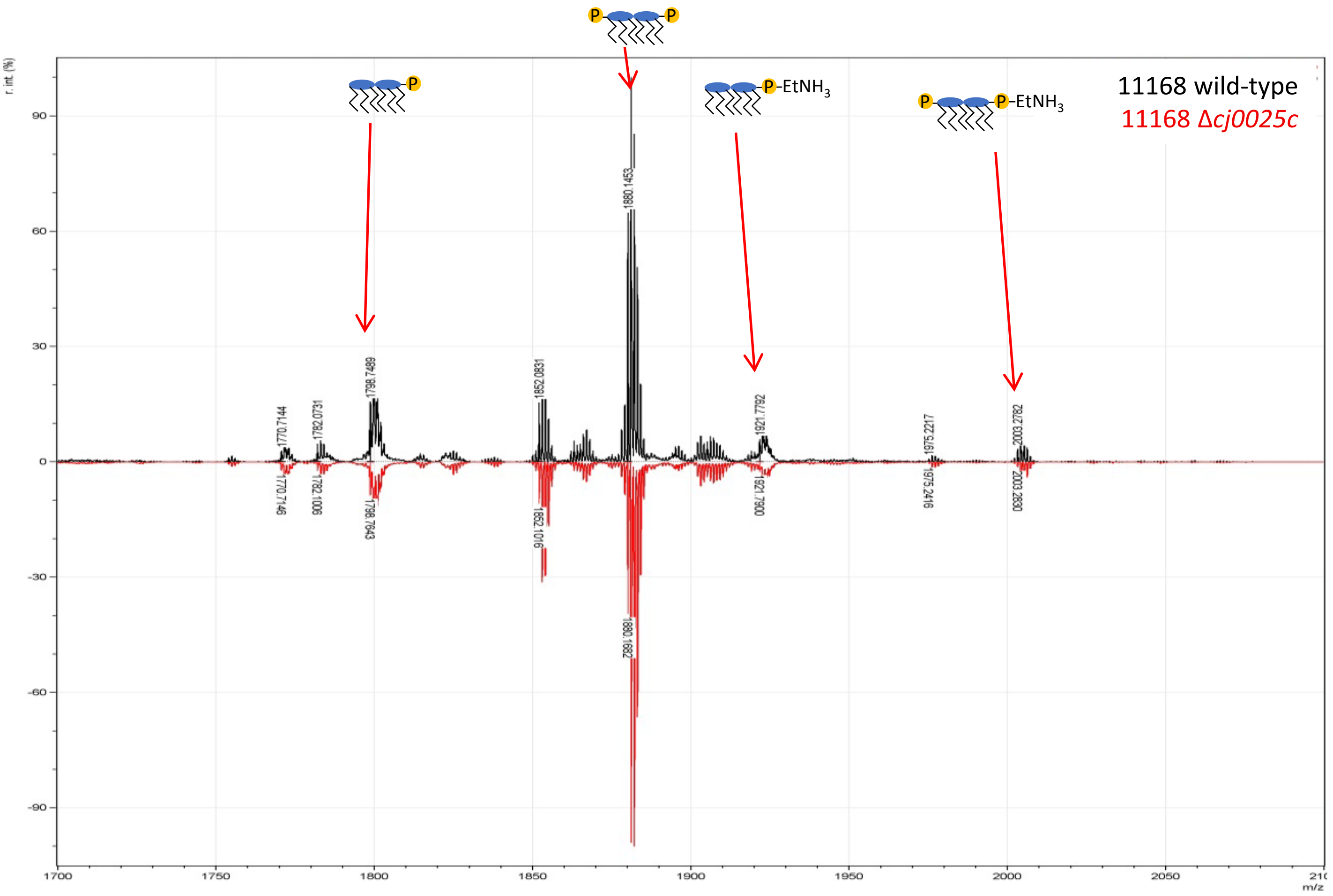

Supplementary Figure S12. MALDI-MS analysis of lipid A from *C. jejuni* NCTC11168 WT (upper, black) and Δ*cj0025c* (lower, red). Major lipid A variants are denoted with arrowheads and structures.

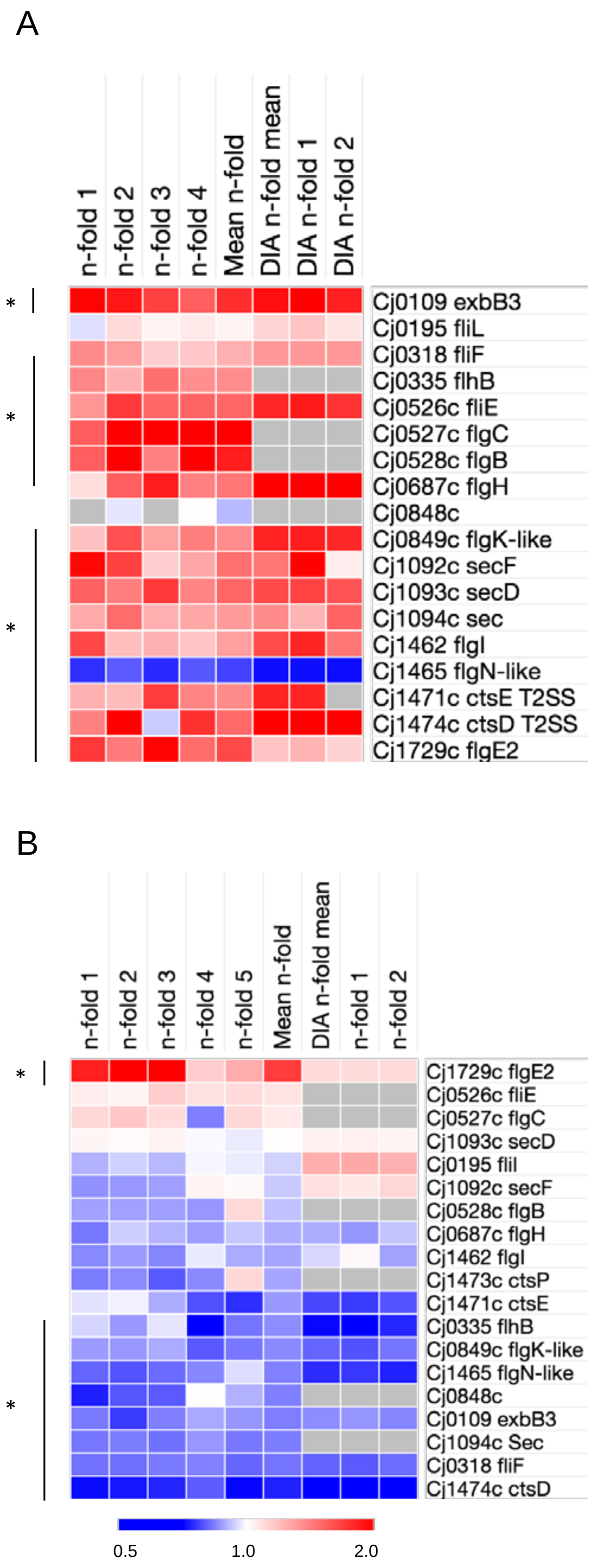

Supplementary Figure S13. Heat maps of proteins associated with secretion of extracellular proteins from (A) *C. jejuni* grown in MH medium compared with MH medium supplemented with 0.1% DOC and (B) comparison of WT and Δ*cj0025c C. jejuni* NCTC11168. *n*=4-5 biological replicates were performed for label-based LC-MS/MS discovery (columns *n*-fold 1-4,5 (left), Mean *n*-fold) and a further 2 biological replicates were used for validation by DIA-SWATH-MS (columns DIA *n*-fold mean and *n*-fold 1-2 (right)). * Proteins deemed significantly changing in abundance.
