# Supplementary material for: Proteomics of Campylobacter jejuni Growth in Deoxycholate Reveals Cj0025c as a Cystine Transport Protein Required for Wild-type Human Infection Phenotypes
Source: Mol Cell Proteomics. 2020 Nov 23;19(8):1263–80. doi: 10.1074/mcp.RA120.002029 (PMC8015009; doi:10.1074/mcp.RA120.002029)
Supplement: Supplementary file 1 [file mmc1.zip › 159411_1_supp_522820_q9nf8k.pdf]

Supplementary Table S1. Instrument settings for targeted metabolomics of amino and organic acids using LC-MS/MS.

Synergi-RP Negative Mode MRM

Name	Q1	Q3	Dwell (ms)	CE	CXP	DP
Glucose/Fucose/Galactose	179	89	100	-20	-15	-45
Lactate	89	43	100	-20	-15	-40
a-Ketoglutatrate/Adipate	145.1	101	100	-20	-15	-45
Citrate	191	111	100	-15	-15	-45
Fumarate	115	71	100	-15	-15	-45
Propionate	73	55	100	-20	-15	-30
Malate	133	115	100	-20	-15	-40
Malonate	103	59	100	-15	-15	-40
Oxaloacetate	131	7	100	-10	-15	-40
Succinate	117	73	100	-20	-15	-40
Pyruvate	87	43	100	-15	-15	-30
Acetyl-CoA	403.6	79	100	-60	-15	-50
Malonyl-CoA	425.6	79	100	-60	-15	-30
Aconitate	173	129	100	-10	-15	-40

Luna Phenyl-Hexyl MRM

Name	Q1	Q3	Dwell (ms)	CE	CXP	DP
Histidine	156	110	100	12	10	93
Homocysteine	136	90	100	10	10	93
Leucine/Isoleucine	132.1	86.2	100	11	10	93
Lysine	147.1	84.1	100	15	10	93
Glutamate	148	84	100	15	10	93
Glutamine	147	130	100	15	10	93
Asparagine	133.1	74	100	17	10	93
Proline	116	70	100	11	10	93
Methionine	150	61	100	30	10	93
Serine	106	60	100	13	10	93
Alanine	90.1	44.2	100	13	10	93
Homoserine	120	44	100	32	10	90
Glycine	76	30	100	18	11.4	78
Tryptophan	205.1	188.3	100	21	12	70
Valine	118	72	100	17	11.99	60
Arginine	175.1	69.8	100	25	10	93
Threonine	120.1	74	100	30	10	93
Argininosuccinate	291.1	70	100	54	13	50
Cysteine	122	76	100	20	10	25
Cystine	241	152	100	20	10	50
Aspartate	134	74	100	21	10	25
Arginine	175.1	70	100	32	10	25
