# Supplementary material for: Proteomics of Campylobacter jejuni Growth in Deoxycholate Reveals Cj0025c as a Cystine Transport Protein Required for Wild-type Human Infection Phenotypes
Source: Mol Cell Proteomics. 2020 Nov 23;19(8):1263–80. doi: 10.1074/mcp.RA120.002029 (PMC8015009; doi:10.1074/mcp.RA120.002029)
Supplement: Supplementary file 1 [file mmc1.zip › 159411_1_supp_522821_q9nf8k.pdf]

Supplementary Table S2. Proteomics of wild-type *C. jejuni* NCTC11168 compared with Δ*cj0025*. Significantly differentially abundant proteins identified by LC-MS/MS; *n*-fold DDA, mean *n*-fold change in Δ*cj0025* / WT *C. jejuni* NCTC11168 across a minimum of 2 label-based biological replicates ($p<0.05$); *n*-fold DIA, mean *n*-fold change in Δ*cj0025* / WT *C. jejuni* NCTC11168 across a minimum of 2 DIA-SWATH MS biological replicates; % Cys/Met, % sulfur-containing amino acid residues within full protein sequence, Cys [cysteine], Met [methionine]. ND, not detected; NQ, not quantified. Bold entries indicate those with functions related to a requirement for sulfur.

**Cj No.**	***Gene***	**Protein**	***n*-fold DDA**	***n*-fold DIA**	**% Cys/Met**	Additional Information
(A) Proteins present at elevated abundance in Δcj0025						
Cj0008	*cj0008*	Putative Uncharacterized Protein	+4.54	ND	1.0% / 0.9%	DUF262-containing protein
Cj0012c	*rrc*	**Non-Haem Iron Protein**	+1.71	+3.59	4.2% / 2.8%	Ferritin-like protein, desulforedoxin-like protein
Cj0030	*cj0030*	Putative Uncharacterized Protein	+3.72	ND	1.7% / 1.1%	PDDEXK4 nuclease domain-containing protein
Cj0033	*cj0033*	Putative Integral Membrane Protein	+4.08	ND	2.5% / 0.7%	Pentapeptide repeat-containing protein
Cj0037c	*cj0037c*	**Putative Cytochrome *c***	+2.72	+8.29	1.4% / 2.9%	Multi-haem cytochrome-like protein
Cj0044c	*cj0044c*	Putative Uncharacterized Protein	+2.17	+6.23	1.1% / 1.0%	Flagellar assembly-like FapA domain protein
Cj0045c	*cj0045c*	**Putative Iron-Binding Protein**	+1.71	ND	1.7% / 2.1%	Haemerythrin-like protein, Fe-S cluster repair-like protein
Cj0057	*cj0057*	**Putative Periplasmic Protein**	+2.33	ND	0.4% / 1.1%	HmcD-like transmembrane redox complex-like protein
Cj0139	*cj0139*	Putative Endonuclease	+2.58	+1.84	0.4% / 1.9%	5-methylcytosine-specific restriction enzyme subunit McrB
Cj0140	*cj0140*	Putative Uncharacterized Protein	+1.51	+1.67	1.6% / 2.7%	5-methylcytosine-specific restriction enzyme subunit McrC
Cj0144	*tlp2*	**Putative Methyl-Accepting Chemotaxis Protein (Transducer-Like Protein Tlp2)**	+2.15	+3.51	0.6% / 0.9%	SAM-dependent; chemotaxis protein, induced by iron
Cj0146c	*trxB*	**Thioredoxin Reductase TrxB**	+1.79	+2.24	2.2% / 3.5%	Thiol-disulfide reductase
Cj0168c	*cj0168c*	Putative Periplasmic Protein	+1.81	ND	0.0% / 1.8%	DUF4148-containing protein
Cj0169	*sodB*	**Superoxide Dismutase [Fe]**	+1.48	+2.06	1.8% / 0.9%	Antioxidant oxidoreductase family protein
Cj0173c	*cfbpC*	Putative Iron-Uptake ABC Transport System ATP-Binding Protein	+1.52	ND	1.3% / 2.3%	Ferric Fe^{3+} uptake-like protein
Cj0175c	*cfbpA*	Putative Iron-Uptake ABC Transport System, Periplasmic Iron-Binding Protein	+1.74	+1.72	0.0% / 2.4%	Ferric Fe^{3+} uptake-like protein
Cj0192c	*clpP*	ATP-Dependent Clp Protease Proteolytic Subunit	+1.58	+1.77	1.5% / 3.1%	Endopeptidase Clp; degrades misfolded proteins
Cj0203	*cj0203*	**Putative Citrate Transporter**	+1.74	ND	1.6% / 3.3%	CitM Na^+/H^+, Na^+/sulfate transporter family; DcuC-like protein
Cj0204	*cptA*	***Campylobacter* Peptide Transporter CptA**	+3.81	ND	0.9% / 3.3%	Oligopeptide transport protein OPT family
Cj0229	*cj0229*	**Putative Acetyltransferase**	+2.04	ND	1.6% / 1.6%	LpxA-like lipid A biosynthesis protein; serine *O*-acetyltransferase-like L-Cys biosynthesis protein
Cj0239c	*cj0239c*	**Nitrogen Fixation Protein NifU**	+1.45	+1.91	3.1% / 3.4%	Fe-S cluster assembly / repair protein
Cj0240c	*iscS*	**Cysteine Desulfurase (NifS Protein Homolog)**	+1.42	+1.76	0.8% / 3.1%	Sulfur liberating protein; Fe-S cluster biosynthesis protein
Cj0256	*eptC*	**Putative Sulfatase Family Protein (Phosphoethanolamine Transferase EptC)**	+1.73	+1.92	1.6% / 1.8%	Arylsulfatase-like; lipid A / *N*-glycan / FlgG modification enzyme
Cj0259	*pyrC*	Dihydroorotase	+2.07	+3.43	3.0% / 3.6%	Pyrimidine metabolism / biosynthesis enzyme
Cj0282c	*serB*	Putative Phosphoserine Phosphatase	+1.66	ND	1.9% / 5.3%	Amino acid metabolism generating free serine
Cj0287c	*greA*	Transcription Elongation Factor GreA	+1.61	+1.80	0.6% / 2.5%	Transcription elongation factor
Cj0297c	*panC*	**Pantothenate Synthetase (Pantoate--Beta-Alanine Ligase)**	+1.80	+1.82	1.8% / 3.9%	CoA biosynthesis enzyme; pathway requires Cys

Cj0298c	*panB*	**3-Methyl-2-Oxobutanoate Hydroxymethyltransferase (Ketopantoate Hydroxymethyltransferase)**	+1.75	+3.24	1.1% / 4.0%	CoA biosynthesis enzyme; pathway requires Cys
Cj0300c	*modC*	**Putative Molybdenum Transport ATP-Binding Protein**	+2.73	ND	0.0% / 1.7%	Sulfate/molybdate transport protein
Cj0303c	*modA*	**Putative Molybdate-Binding Lipoprotein**	+3.53	ND	0.4% / 0.8%	Molybdate transport protein
Cj0304c	*bioC*	**Malonyl-[Acyl Carrier Protein] O-Methyltransferase**	+1.64	ND	1.3% / 1.8%	Biotin synthesis protein; SAM-dependent methyltransferase
Cj0305c	*cj0305c*	**Putative Uncharacterized Protein**	+3.66	ND	1.0% / 1.0%	DUF452-containing protein; biotin biosynthesis protein BioG
Cj0306c	*bioF*	**8-Amino-7-Oxononanoate Synthase**	+3.36	ND	1.6% / 1.6%	Biotin biosynthesis protein BioF
Cj0358	*cj0358*	**Putative Cytochrome C551 Peroxidase**	+1.62	+2.27	1.2% / 1.5%	Di-haem-type, iron-binding cytochrome *c*-like peroxidase
Cj0379c	*msrP*	**Protein-Methionine-Sulfoxide Reductase Catalytic Subunit MsrP Precursor**	+1.93	+3.34	0.3% / 3.0%	Molybdopterin-binding protein; redox repair protein
Cj0381c	*pyrF*	Orotidine 5'-Phosphate Decarboxylase	+1.99	ND	2.5% / 2.5%	Pyrimidine biosynthesis enzyme
Cj0414	*cj0414*	**Putative Oxidoreductase Subunit**	+2.96	+4.61	0.4% / 4.5%	Gluconate 2-dehydrogenase, oxidoreductase-like enzyme
Cj0415	*cj0415*	**Putative GMC Oxidoreductase Subunit**	+2.97	+4.53	1.4% / 3.3%	FAD flavoprotein-like oxidoreductase
Cj0419	*cj0419*	Putative Histidine Triad (HIT) Family Protein	+2.17	+1.57	2.5% / 3.3%	Putative nucleotide-binding protein
Cj0420	*cj0420*	Putative Periplasmic Protein	+2.64	+2.05	0.0% / 2.1%	YceI-like lipid-binding protein
Cj0435	*fabG*	**3-Oxoacyl-[Acyl Carrier Protein] Reductase**	+1.36	+2.30	1.2% / 2.8%	NAD/NADP-dependent oxidoreductase; fatty acid biosynthesis, biotin metabolism
Cj0436	*cj0436*	**Putative Pyridoxamine 5'-Phosphate Oxidase**	+3.41	ND	2.9% / 3.7%	Ferredoxin reductase FMN-binding domain protein
Cj0453	*thiC*	**Phosphomethylpyrimidine Synthase**	+1.57	+1.96	1.9% / 4.9%	Thiamine biosynthesis protein ThiC, SAM-dependent process
Cj0465c	*ctb*	**Group 3 Truncated Hemoglobin Ctb**	+1.45	+1.55	0.0% / 4.7%	Cytochrome peroxidase and/or oxygen detoxification
Cj0480c	*cj0480c*	Putative Transcriptional Regulator	+2.14	ND	2.0% / 2.0%	ArsR/IclR-like transcriptional regulator
Cj0481	*dapA*	Putative Dihydropicolinate Synthase	+4.66	ND	0.7% / 2.3%	Putative *N*-acetylneuraminate lyase; generates pyruvate from fucose
Cj0485	*cj0485*	**Putative Oxidoreductase**	+3.16	ND	1.1% / 2.3%	L-fucose dehydrogenase-like protein; part of fucose uptake cluster
Cj0509c	*clpB*	Chaperone Protein ClpB	+1.48	+1.42	0.0% / 2.1%	DnaK-associated refolding protein
Cj0520	*cj0520*	Putative Membrane Protein	+1.67	ND	0.7% / 2.0%	DUF883-containing protein
Cj0597	*fba*	Fructose-Bisphosphate Aldolase	+1.87	+1.85	1.1% / 1.1%	Gluconeogenesis pathway protein
Cj0618	*cj0618*	Putative Uncharacterized Protein	+1.68	ND	1.4% / 0.9%	DUF2920-containing protein; motility-associated protein
Cj0628	*capA*	Putative Lipoprotein (*Campylobacter* Adhesion Protein A CapA)	+3.68	ND	0.2% / 1.2%	Phase variable autotransporter; putative adherence factor
Cj0632	*ilvC*	Ketol Acid Reductoisomerase (Acetohydroxy-Acid Isomeroreductase)	+1.49	+1.53	1.2% / 3.5%	Branched chain amino acid biosynthesis protein
Cj0664c	*rplI*	Ribosomal Protein L9	+1.73	+1.80	0.7% / 0.7%	Ribosomal-associated translation protein
Cj0690c	*cj0690c*	**Putative Restriction / Modification Enzyme**	+3.13	ND	0.5% / 1.0%	SAM-dependent RNA/DNA methyltransferase
Cj0692c	*cj0692c*	Putative Membrane Protein	+1.43	+1.83	0.0% / 1.3%	No known functional annotation
Cj0708	*cj0708*	Putative Ribosomal Pseudouridine Synthase	+1.98	+1.79	1.2% / 2.0%	RluA/RsuA-family pseudouridine synthase-like protein
Cj0715	*cj0715*	5-Hydroxyisourate Hydrolase	+3.53	+10.09	0.0% / 0.8%	Purine catabolism enzyme
Cj0719c	*cj0719c*	Pyridoxal Phosphate (PLP) Homeostasis Protein	+2.73	ND	2.4% / 2.4%	PLP-binding, PLP chaperone-like protein
Cj0760	*cj0760c*	**Putative Uncharacterized Protein**	+3.45	ND	0.3% / 1.5%	Thioesterase-like protein
Cj0772c	*cj0772c*	**Putative NLPA Family Lipoprotein**	+1.73	+1.56	0.0% / 0.8%	D-methionine permease-like protein
Cj0778	*peb2*	**Major Antigenic Peptide PEB2**	+2.38	+2.33	0.0% / 2.0%	Thiosulfate/sulfate-binding protein homolog
Cj0779	*tpx*	**Thiol Peroxidase**	+1.50	+2.01	3.4% / 2.3%	Thioredoxin-like oxidative stress protein
Cj0820c	*fliP*	Flagellar Biosynthesis Protein FliP	+1.50	ND	0.0% / 5.7%	Flagellar structural protein, type III secretion FliP-like protein

Cj0836	*ogt*	**Methylated-DNA—Protein-Cysteine Methyltransferase**	+2.26	ND	2.7% / 2.0%	SAM-dependent methyltransferase, methylated DNA repair protein
Cj0899c	*thiJ*	**4-Methyl-5(Beta-Hydroxyethyl)-Thiazole Monophosphate Synthesis Protein**	+1.68	+1.74	2.6% / 2.1%	Thiamine biosynthesis protein
Cj0914c	*ciaB*	*Campylobacter* Invasion Antigen B CiaB	+1.42	+2.14	1.0% / 1.8%	Virulence-associated, invasion-specific protein
Cj0977	*cj0977*	**Putative Uncharacterized Protein**	+1.77	ND	1.0% / 2.1%	Thioesterase / thioester dehydrogenase-like protein
Cj0983	*jlpA*	Uncharacterized Lipoprotein Cj0983 Precursor	+1.37	+1.40	0.8% / 1.2%	*Campylobacter* adhesion protein JlpA
Cj0998c	*cj0998c*	Putative Periplasmic Protein	+1.74	+1.94	0.5% / 2.1%	No known functional associations
Cj1000	*cj1000*	Putative Transcriptional Regulator (LysR Family)	+1.43	+2.36	0.3% / 1.0%	LysR substrate-binding type transcriptional regulator
Cj1004	*cj1004*	Putative Periplasmic Protein	+1.64	ND	0.0% / 0.7%	Beta-lactamase inhibitor-like protein
Cj1041c	*cj1041c*	Putative Periplasmic ATP/GTP-Binding Protein	+1.48	+1.65	0.0% / 2.1%	TolB-like protein
Cj1050c	*cobB*	NAD-Dependent Protein Deacylase	+1.51	+1.51	3.0% / 2.6%	Bacterial sirtuin-like protein CobB
Cj1051c	*cjeI*	**Restriction Modification Enzyme**	+3.24	ND	1.2% / 1.5%	SAM-dependent methyltransferase; type I restriction enzyme R-like
Cj1066	*rdxA*	**Nitroreductase**	+1.85	+3.64	2.5% / 2.0%	NfsB-like flavin-associated oxidoreductase
Cj1070	*rpsF*	30S Ribosomal Protein S6	+1.47	ND	0.0% / 1.6%	Ribosomal-associated translation protein
Cj1082c	*thiD*	**Phosphomethylpyrimidine Kinase**	+1.50	+1.64	3.0% / 3.0%	Thiamine biosynthesis enzyme
Cj1083c	*cj1083c*	Putative Nuclease	+1.73	ND	1.3% / 0.4%	DNA glycosylase, DNA oxidative damage repair-like protein
Cj1084c	*cj1084c*	Putative ATP/GTP-Binding Protein	+1.54	+1.38	0.8% / 1.2%	DUF815-containing protein
Cj1087c	*cj1087c*	Putative Peptidase	+2.67	+2.67	0.0% / 1.7%	Peptidase M23 family protein
Cj1110c	*tlp8*	**Putative MCP-Type Signal Transduction-Like Protein**	+1.98	+2.70	0.9% / 2.8%	Transducer-like protein Tlp8; SAM-dependent energy taxis protein
Cj1136	*cj1136*	Putative Glycosyltransferase	+2.05	ND	1.5% / 1.8%	Putative phase variable sugar transferase from nucleotide-sugar
Cj1137c	*cj1137c*	Putative Glycosyltransferase	+2.86	ND	1.5% / 2.4%	Putative phase variable sugar transferase from nucleotide-sugar
Cj1138	*cj1138*	Putative Glycosyltransferase	+2.64	ND	1.3% / 1.0%	Putative phase variable sugar transferase from nucleotide-sugar
Cj1140	*cstIII*	Alpha-2,3 Sialyltransferase	+3.91	ND	2.0% / 1.7%	Sialyltransferase CMP-NeuAc to other glycoconjugate-like protein
Cj1141	*neuB1*	Sialic Acid Synthase (*N*-Acetylneuraminic Acid Synthetase)	+3.12	ND	1.5% / 3.5%	Sialic acid biosynthesis-like protein
Cj1142	*neuC1*	Putative UDP-*N*-Acetylglucosamine 2-Epimerase	+3.43	ND	0.8% / 2.4%	Nucleotide-sugar epimerase-like protein
Cj1143	*cgtA*	Two-Domain Bifunctional Protein (Beta-1,4-*N*-Acetylgalactosaminyltransferase/CMP-Neu5Ac Synthase)	+2.27	ND	1.3% / 1.7%	Sugar biosynthesis bifunctional protein
Cj1149c	*gmhA*	Phosphoheptose Isomerase 1	+1.64	+1.51	3.2% / 2.7%	Lipooligosaccharide (LOS) biosynthesis protein
Cj1150c	*hldE*	Bifunctional Protein HldE (D-Beta-D-Heptose 7-Phosphate Kinase; D-Beta-D-Heptose 1-Phosphate Adenylyltransferase)	+1.61	+1.46	1.5% / 0.9%	WaaE-like LOS biosynthesis protein
Cj1180c	*cj1180c*	Putative ABC Transporter ATP-Binding Protein	+1.46	+1.51	2.4% / 0.9%	LolD-like lipoprotein-releasing ATP-binding protein
Cj1183c	*cfa*	**Cyclopropane-Fatty-Acyl-Phospholipid Synthase**	+2.96	+5.74	1.0% / 3.1%	SAM-dependent methyltransferase class I
Cj1191c	*cetC/aer1*	Putative PAS Domain Containing Signal Transduction Sensor Protein	+2.20	+3.26	0.6% / 2.4%	Methyl-accepting, energy taxis chemotaxis-like protein
Cj1201	*metE*	**5-Methyltetrahydropteroyltriglutamate--Homocysteine Methyltransferase (Cobalamin-Independent Methionine Synthase)**	+1.77	+3.77	0.6% / 0.6%	Methionine biosynthesis protein
Cj1219c	*cj1219c*	Putative Periplasmic Protein	+1.86	+1.89	0.1% / 1.2%	Branched chain amino acid transporter-like protein
Cj1220	*groS*	10 kDa Chaperonin GroES	+1.64	+2.58	0.0% / 1.2%	Chaperone / re-folding protein
Cj1221	*groL*	60 kDa Chaperonin GroEL	+1.71	+3.05	0.2% / 3.3%	Chaperone / re-folding protein
Cj1228c	*htrA*	Periplasmic Serine Endorotease DegP-Like HtrA (Protease DO)	+1.71	+3.48	0.0% / 0.6%	HtrA (protease DO), protease-like degradation protein
Cj1235	*cj1235*	Putative Peptidase M23 Family Protein	+1.49	+1.75	0.0% / 0.4%	Zinc metallopeptidase-like protein
Cj1237c	*cj1237c*	Putative Phosphatase	+1.80	+1.81	1.9% / 2.2%	Ppx/GppA-type exophosphatase-like protein

Cj1240c	*cj1240c*	Putative Periplasmic Protein	+2.53	ND	2.0% / 3.5%	No known functional associations
Cj1242	*ciaC*	*Campylobacter* Invasion Antigen C (CiaC)	+1.73	ND	0.0% / 1.9%	Secreted, invasion-associated virulence factor
Cj1266c	*hydB*	**Ni/Fe Hydrogenase Large Subunit HydB**	+1.50	+1.57	1.9% / 1.9%	NADH quinone oxidoreductase-like protein
Cj1279c	*flpA*	Putative Fibronectin Domain-Containing Lipoprotein	+1.42	+1.65	0.2% / 1.9%	*Campylobacter* fibronectin-binding, adherence protein
Cj1295	*cj1295*	Putative Uncharacterized Protein	+2.57	ND	2.3% / 1.8%	Putative phase variable, DUF2172-containing protein
Cj1299	*acpP2*	**Putative Acyl Carrier Protein**	+4.73	ND	1.3% / 2.6%	Fatty acid biosynthesis acyl-transfer protein
Cj1300	*cj1300*	**Putative SAM Domain-Containing Methyltransferase**	+3.30	ND	2.0% / 2.0%	SAM-dependent methyltransferase
Cj1301	*cj1301*	**Putative Uncharacterized Protein**	+6.22	ND	3.6% / 3.6%	Dioxygenase-like protein, vicinal oxygen chelate family
Cj1309c	*cj1309c*	**Putative Uncharacterized Protein**	+3.31	ND	0.6% / 2.5%	Putative phase variable, acyl carrier protein
Cj1319	*cj1319*	Putative Nucleotide Sugar Dehydratase	+4.41	ND	1.5% / 1.9%	Putative phase variable, NAD-dependent 4,6-dehydratase LegB-like protein
Cj1320	*cj1320*	Putative Aminotransferase (DegT Family)	+3.49	ND	2.9% / 0.8%	Putative phase variable, pyridoxal phosphate-dependent transferase
Cj1324	*cj1324*	Putative Uncharacterized Protein	+4.10	ND	2.9% / 2.9%	*N*-acetyl sugar amidotransferase-like protein
Cj1325	*cj1325*	**Putative Methyltransferase**	+2.89	ND	0.9% / 2.2%	SAM-dependent methyltransferase
Cj1327	*legI/neuB2*	*N*,*N*′-Diacetyllegionaminic Acid Synthase	+4.20	ND	1.8% / 2.4%	Legionaminic acid biosynthesis protein
Cj1328	*legG/neuC2*	GDP/UDP-*N*,*N*′-Diacetylbacillosamine 2-Epimerase (Hydrolyzing)	+2.52	ND	2.1% / 2.6%	Legionaminic acid biosynthesis protein
Cj1329	*cj1329*	**Putative Sugar-Phosphate Nucleotide Transferase**	+4.90	ND	0.6% / 1.8%	Putative SAM-binding, cystathione beta synthase domain protein
Cj1330	*cj1330*	**Putative Uncharacterized Protein**	+4.34	ND	1.3% / 0.3%	Gfo/Idh/MocA oxidoreductase-family, flagellin modification protein PtmF
Cj1331	*legF / ptmB*	CMP-*N*,*N*′-Diacetyllegionaminic Acid Synthase	+3.93	ND	1.7% / 2.1%	Legionaminic acid biosynthesis, flagellin modification protein
Cj1332	*ptmA*	**Putative Oxidoreductase**	+5.93	ND	2.7% / 2.3%	Flagellin modification, NAD(P)-dependent oxidoreductase
Cj1333	*pseD*	PseD Protein	+1.94	NQ	0.6% / 2.1%	Motility accessory factor, DUF115-containing protein
Cj1334	*maf3*	Putative Uncharacterized Protein Maf3	+1.98	ND	1.5% / 1.1%	Motility accessory factor, DUF115-containing protein
Cj1337	*pseE*	PseE Protein	+3.74	ND	1.1% / 1.6%	Motility accessory factor, DUF115-containing protein
Cj1338c	*flaB*	Flagellin B	+1.72	+1.58	0.0% / 1.6%	Major flagellar subunit
Cj1341c	*maf6*	Putative Uncharacterized Protein Maf6	+2.63	NQ	1.8% / 2.0%	Motility accessory factor, DUF115-containing protein
Cj1342c	*maf7*	Putative Uncharacterized Protein Maf7	+2.74	ND	1.9% / 3.1%	Motility accessory factor, esterase-like protein, DUF2920-containing protein
Cj1369	*cj1369*	Putative Permease	+2.01	ND	0.9% / 3.9%	Xanthine/uracil/vitamin C-like permease
Cj1370	*cj1370*	Putative Nucleotide Phosphoribosyltransferase	+1.37	+1.88	0.0% / 1.4%	Nicotinate/uracil phosphoribsyltransferase-like protein
Cj1372	*cj1372*	Putative Periplasmic Protein	+1.82	ND	0.0% / 2.1%	Toluene tolerance-like protein
Cj1376	*cj1376*	Putative Periplasmic Protein	+4.03	ND	1.1% / 1.5%	Esterase domain-containing protein
Cj1380	*cj1380*	**Putative Periplasmic Protein**	+1.46	+1.66	1.3% / 1.3%	Thioredoxin-like protein, thiol:disulfide interchange protein DsbC
Cj1381	*cj1381*	Putative Lipoprotein	+1.53	ND	1.1% / 2.8%	Bacterial surface antigen motif-containing protein
Cj1382c	*fldA*	**Flavodoxin**	+1.49	+2.00	1.1% / 2.5%	Flavoprotein superfamily protein
Cj1404	*nadD*	Probable Nicotinate-Nucleotide Adenyltransferase	+1.48	+1.60	1.1% / 1.1%	NAD(+) biosynthesis-associated protein
Cj1405	*rsfS*	Ribosomal Silencing Factor RsfS	+1.42	+1.93	0.0% / 0.9%	Translation repression-associated protein
Cj1421c	*cj1421c*	Putative Sugar Transferase	+2.61	ND	1.0% / 1.1%	Putative phase variable, DUF2972-containing protein
Cj1422c	*cj1422c*	Putative Sugar Transferase	+3.51	ND	1.8% / 1.6%	Putative phase variable, DUF2972-containing protein

Cj1426c	*cj1426c*	**Putative Methyltransferase Family Protein**	+3.56	ND	2.1% / 2.1%	Putative phase variable, SAM-dependent methyltransferase
Cj1427c	*cj1427c*	Putative Sugar-Nucleotide Epimerase/Dehydratase	+3.97	ND	1.6% / 3.2%	Putative phase variable, NAD-dependent enzyme
Cj1428c	*fcl*	GDP-L-Fucose Synthase	+3.44	ND	0.9% / 3.2%	Nucleotide-sugar biosynthesis protein, NAD-dependent enzyme
Cj1429c	*cj1429c*	**Putative Uncharacterized Protein**	+3.29	ND	1.9% / 2.6%	Putative methyltransferase-like protein 24
Cj1430c	*rfbC*	Putative dTDP-4-Dehydrorhamnose 3,5-Epimerase	+1.96	ND	1.1% / 1.7%	Polysaccharide biosynthesis-like protein
Cj1431c	*hddC*	Capsular Polysaccharide Heptosyltransferase	+3.43	ND	1.2% / 1.5%	Capsule biosynthesis CapA-like protein
Cj1432c	*cj1432c*	Putative Sugar Transferase	+3.68	ND	1.3% / 2.3%	Nucleotide-sugar glycosyltransferase-like protein
Cj1433c	*cj1433c*	**Putative Uncharacterized Protein**	+6.29	ND	3.5% / 1.4%	Radical SAM, SAM-dependent molybdenum cofactor biosynthesis protein
Cj1434c	*cj1434c*	Putative Sugar Transferase	+3.05	ND	2.0% / 2.2%	Nucleotide-sugar glycosyltransferase-like protein
Cj1435c	*cj1435c*	Putative Phosphatase	+3.49	ND	1.4% / 1.4%	HAD domain-containing protein, phosphoserine phosphatase-like protein
Cj1436c	*cj1436c*	**Aminotransferase**	+3.39	ND	0.8% / 2.3%	Pyridoxal phosphate-dependent enzyme, Cys desulfurase-like
Cj1437c	*cj1437c*	**Aminotransferase**	+3.60	ND	1.9% / 1.6%	Pyridoxal phosphate-dependent enzyme, Cys desulfurase-like
Cj1438c	*cj1438c*	Putative Sugar Transferase	+4.27	ND	1.4% / 1.9%	Polysaccharide biosynthesis, TupA-like protein
Cj1439c	*glf*	UDP-Galactopyranose Mutase	+3.37	ND	1.6% / 1.6%	Nucleotide-sugar glycosyltransferase-like flavoprotein
Cj1440c	*cj1440c*	Putative Sugar Transferase	+1.98	ND	2.5% / 2.2%	Nucleotide-sugar glycosyltransferase-like protein
Cj1441c	*kfiD*	UDP-Glucose 6-Dehydrogenase	+3.26	ND	0.3% / 1.3%	NAD-dependent oxidoreductase process
Cj1491c	*cj1491c*	Putative Two-Component Regulator	+3.73	ND	0.2% / 2.3%	DNA-binding response regulator-like protein
Cj1508c	*fdhD*	**Sulfur Carrier Protein FdhD**	+1.71	+3.33	3.1% / 3.5%	Formate dehydrogenase, sulfur carrier from IcsS to molybdenum
Cj1513c	*cj1513c*	Possible Periplasmic Protein	+3.90	ND	0.0% / 1.6%	Possible TAT translocation pathway protein
Cj1516	*cj1516*	**Putative Periplasmic Oxidoreductase**	+1.50	+2.47	0.2% / 3.9%	Multi-copper oxidase-like, cupredoxin-like protein
Cj1517	*moaD*	**Putative Molybdopterin Converting Factor Subunit 1**	+1.44	+2.60	2.7% / 1.4%	Sulfur carrier ThiS-like protein, sulfur transfer protein
Cj1522c	*cas1*	CRISPR-Associated Endonuclease Cas1	+2.81	ND	2.0% / 0.7%	Defence against foreign DNA-like protein
Cj1523c	*cas9*	CRISPR-Associated Endonuclease Cas9	+3.71	ND	0.7% / 1.0%	Defence against foreign DNA-like protein
Cj1540	*tupA*	Tungstate-Binding Protein TupA Precursor	+1.68	+1.64	0.7% / 2.2%	Solute-binding protein
Cj1549c	*hsdR*	Type I Restriction Enzyme R Protein HsdR	+2.80	ND	0.8% / 2.1%	ATPase, nuclease-like protein
Cj1550c	*rloH*	Putative ATP/GTP-Binding Protein	+3.97	ND	0.9% / 2.6%	ABC transporter-like ATP-binding protein
Cj1551c	*hsdS*	**Putative Type I Restriction Enzyme S Protein HsdS**	+2.76	ND	1.3% / 2.4%	SAM-dependent methylase
Cj1552c	*mloB*	Putative Uncharacterized Protein	+3.31	ND	0.8% / 2.5%	DNA-binding helicase, putative transcriptional regulator
Cj1553c	*hsdM*	**Putative Type I Restriction Enzyme M Protein HsdM**	+4.09	ND	1.2% / 1.4%	SAM-dependent methyltransferase
Cj1555c	*cj1555c*	**Putative Uncharacterized Protein**	+3.57	ND	1.9% / 2.8%	Putative NAD/NADP oxidoreductase / flavin reductase
Cj1556	*cj1556*	Putative Transcriptional Regulator	+2.92	ND	0.9% / 0.9%	Transcriptional regulator, oxidative and aerobic stress
Cj1585c	*cj1585c*	**Putative Oxidoreductase**	+4.11	ND	3.1% / 1.0%	Fe-S cluster, ferredoxin-like putative D-lactate dehydrogenase
Cj1586	*cgb*	**Single Domain Haemoglobin**	+2.18	+5.80	1.4% / 4.3%	Haem-containing protein, flavoprotein-like protein
Cj1602	*cj1602*	Putative Uncharacterized Protein	+2.97	ND	0.9% / 0.9%	Putative restriction endonuclease-like protein HgrA
Cj1625c	*sdaC*	L-Serine Amino Acid Transporter	+1.62	ND	1.2% / 1.9%	Serine transport protein SdaC
Cj1626c	*cj1626c*	Putative Periplasmic Protein	+2.29	+1.82	0.0% / 3.6%	Inhibitor-like protein
Cj1653	*cj1653*	Putative Lipoprotein	+2.48	ND	1.0% / 1.8%	Cysteine peptidase-like protein

Cj1663	*cj1663*	Putative ABC Transport System ATP-Binding Protein	+1.40	+1.82	0.5% / 2.8%	Periplasmic transport-like protein
Cj1665	*cj1665*	**Putative Lipoprotein Thioredoxin**	+1.53	ND	1.8% / 1.8%	Thioredoxin family-like protein
Cj1668c	*cj1668c*	Putative Periplasmic Protein	+3.59	ND	1.4% / 1.4%	UreG (urease) accessory, CobW cobalamin synthesis-like protein
Cj1679	*cj1679*	Putative Uncharacterized Protein	+4.39	ND	1.5% / 2.1%	TPR repeat-containing protein
Cj1721c	*cj1721c*	Putative Outer Membrane Protein	+2.37	ND	0.0% / 0.9%	Outer membrane porin-like protein
Cj1726c	*metA*	**Homoserine *O*-Succinyltransferase**	+2.18	+2.61	0.3% / 2.7%	Cys/Met metabolism enzyme, acetyl-CoA-dependent enzyme
Cj1727c	*metB*	**Putative *O*-Acetylhomoserine(Thiol) Lyase**	+1.96	+4.91	0.7% / 0.5%	Cys/Met metabolism enzyme
Cj1731c	*ruvC*	Crossover Junction Endodeoxyribonuclease RuvC	+2.31	ND	0.0% / 1.6%	Holliday junction resolvase
(B) Proteins present at significantly reduced abundance in Δcj0025						
Cj0007	*gltB*	**Glutamate Synthase (NADPH) Large Subunit**	-1.60	-1.64	1.0% / 2.5%	Ferredoxin-dependent, Fe-S flavoprotein
Cj0010c	*rnhB*	Ribonuclease HII (RNase II)	-1.56	ND	2.6% / 2.6%	RNA endonuclease
Cj0021c	*cj0021c*	Putative Fumarylacetoacetate (FAA) Hydrolase Family Protein	-2.14	-17.31	1.7% / 2.1%	Phe/Tyr degrading, fumarate-yielding enzyme
Cj0025c	*cj0025c*	Putative Sodium:Dicarboxylate Family Transmembrane Symporter	-1.90	-38.05	1.1% / 4.8%	*Deleted gene*
Cj0029	*ansA*	Cytoplasmic L-Asparaginase	-1.53	-6.82	0.6% / 1.8%	Asp generating asparaginase
Cj0035	*cj0035*	Bcr/CflA Family Efflux Transporter	-2.34	ND	1.5% / 3.8%	Antibiotic efflux-like, drug resistance-associated transporter
Cj0068	*pspA*	**Protease**	-1.36	-1.54	0.3% / 1.3%	Biotin-dependent acetyl-coA carboxylase-like, ClpP-like proteolytic domain-containing protein
Cj0082	*cydB*	**Cytochrome bd Oxidase Subunit II**	-1.57	ND	0.8% / 2.9%	Cytochrome ubiquinol oxidase subunit
Cj0089	*cj0089*	Putative Lipoprotein	-1.54	-1.82	0.9% / 2.0%	Tetricopeptide-like helical domain protein
Cj0090	*cj0090*	Putative Lipoprotein	-1.89	ND	0.8% / 1.6%	YflR-like lipoprotein, DUF1425-containing protein
Cj0093	*cj0093*	Putative Periplasmic Protein	-1.60	-2.56	0.5% / 1.2%	CsgG Curli domain; LptE lipopolysaccharide assembly-like protein
Cj0098	*fmt*	**Methionyl-tRNA Formyltransferase**	-1.74	-2.39	2.3% / 1.6%	Met-tRNA modification, initiation protein
Cj0102	*atpF*	ATP Synthase F0 Sector B' Subunit	-1.64	-1.61	0.0% / 5.7%	Component of transmembrane proton gradient forming ATPase
Cj0109	*exbB3*	Putative MotA/TolQ/ExbB Proton Channel Family Protein	-1.44	-1.37	1.1% / 0.5%	Flagellar motor protein, biopolymer transport-like protein
Cj0110	*exbD3*	**Putative ExbD/TolR Family Transport Protein**	-1.37	-1.56	0.0% / 2.3%	Ferric iron uptake-like transport protein
Cj0119	*cj0119*	**Putative Hydrolase**	-3.50	-21.15	1.7% / 1.2%	Cysteine hydrolase-like, isochorismatase / nicotinamidase
Cj0120	*cj0120*	Putative Uncharacterized Protein	-1.72	ND	2.5% / 2.0%	DNA recombination repair RecO-like protein
Cj0125c	*cj0125c*	**Putative Uncharacterized Protein**	-1.59	ND	3.3% / 0.8%	DnaK suppressor-like, 4 Cys zinc finger-like protein
Cj0135	*cj0135*	**Putative Uncharacterized Protein**	-1.57	ND	5.9% / 3.5%	DUF448-containing, YlxR-like, 4 Cys zinc finger-like protein
Cj0154c	*rsmI*	**Ribosomal RNA Small Subunit Methyltransferase I**	-2.01	ND	1.5% / 1.5%	SAM-dependent methyltransferase
Cj0158c	*cj0158c*	**Putative Haem-Binding Lipoprotein**	-1.52	NQ	2.1% / 0.7%	Cytochrome *c* oxidase Cbb3-like protein
Cj0160c	*queE*	**7-Carboxy-7-Deazaguanine Synthase QueE**	-1.55	NQ	2.4% / 2.0%	Fe-S cluster binding protein, SAM-dependent process
Cj0161c	*moaA*	**GTP 3'-8-Cyclase (Cyclic Pyranopterin Monophosphate Synthase)**	-1.38	-1.48	1.9% / 1.2%	Molybdenum cofactor biosynthesis protein A
Cj0170	*cj0170*	**Putative Uncharacterized Protein**	-3.73	-18.33	1.6% / 2.8%	Putative class I SAM-dependent methyltransferase
Cj0190c	*cj0190c*	Putative Uncharacterized Protein	-1.41	-2.02	2.0% / 2.4%	Mg-chelatase-related protein, YfiB-like protein
Cj0198c	*cj0198c*	Helicase-Like Protein	-1.51	-1.93	0.3% / 1.8%	Replication associated recombination-like, MgsA replication-like
Cj0199c	*cj0199c*	Putative Periplasmic Protein	-2.31	-2.78	0.3% / 1.3%	No known functional associations

Cj0224	*argC*	*N*-Acetyl-Gamma-Glutamyl-Phosphate Reductase	-1.56	-1.57	1.5% / 1.5%	Arg biosynthesis from Glu; amino acid metabolism/biosynthesis
Cj0226	*argB*	Acetylglutamate Kinase	-1.57	-1.61	2.5% / 2.5%	Arg biosynthesis from Glu; amino acid metabolism/biosynthesis
Cj0227	*argD*	Acetylornithine Aminotransferase	-1.52	-1.78	2.3% / 1.5%	Arg biosynthesis from Glu; amino acid metabolism/biosynthesis
Cj0238	*cj0238*	Putative Mechanosensitive Ion Channel Family Protein	-1.56	-1.57	0.8% / 1.4%	MscS-like protein
Cj0249	*cj0249*	**Putative Uncharacterized Protein**	-1.66	ND	3.8% / 1.9%	Putative cysteine permease-like protein
Cj0250c	*cj0250c*	Putative MFS (Major Facilitator Superfamily) Transport Protein	-1.53	ND	2.3% / 3.4%	Solute transport-like protein
Cj0252	*moaC*	**Cyclic Pyranopterin Monophosphate Synthase Accessory Protein**	-2.13	ND	1.9% / 5.7%	SAM-dependent, molybdenum cofactor biosynthesis protein C
Cj0263	*zupT*	Zinc Transporter ZupT	-1.62	ND	0.7% / 2.4%	Zinc uptake permease
Cj0289c	*peb3*	**Major Antigenic Peptide PEB3**	-2.22	-5.72	0.4% / 1.2%	Periplasmic sulfate/thiosulfate transport protein
Cj0316	*pheA*	Chorismate Mutase / Prephenate Dehydratase	-1.40	-1.84	1.1% / 1.7%	Phe/Tyr/Trp amino acid biosynthesis
Cj0318	*fliF*	Flagellar M-Ring Protein	-1.49	-1.58	0.0% / 1.2%	Flagellar export apparatus, flagellar basal body protein
Cj0319	*fliG*	Flagellar Motor Switch Protein	-1.47	-1.45	0.0% / 3.8%	Flagellar directional motor protein
Cj0333c	*fdxA*	**Ferredoxin**	-1.55	ND	9.6% / 2.1%	Fe-S cluster protein
Cj0335	*flhB*	Flagellar Biosynthetic Protein FlhB	-1.37	-2.41	0.3% / 4.1%	Flagellar export apparatus with FliI, FliH
Cj0339	*cj0339*	Major MFS (Major Facilitator Superfamily) Transport Protein	-1.51	-1.40	0.9% / 4.6%	Metabolite : H^+ symporter
Cj0347	*trpF*	*N*-(5'-Phosphoribosyl)-Anthranilate Isomerase (PRAI)	-1.48	-1.69	1.0% / 0.5%	Trp amino acid biosynthesis
Cj0353c	*cj0353c*	Phosphatase	-1.39	-1.55	2.1% / 1.2%	Ppx/GppA-like phosphatase
Cj0356c	*folB*	Putative Dihydroneopterin Aldolase	-1.56	ND	1.0% / 1.0%	Tetrahydrofolate biosynthesis, purine/pyrimidine biosynthesis
Cj0363c	*hemN*	**Putative Oxygen-Independent Coproporphyrinogen III Oxidase**	-1.40	-1.41	1.8% / 2.7%	Radical SAM, Fe-S centre protein
Cj0374	*cj0374*	**UPF0234 Protein Cj0374**	-1.46	-1.75	0.6% / 1.8%	DUF520-containing, YajQ-like, ferredoxin-like protein
Cj0387	*aroK*	Shikimate Kinase	-1.47	-1.71	1.8% / 2.4%	Phe/Tyr/Trp amino acid biosynthesis
Cj0395c	*cj0395c*	Putative Uncharacterized Protein	-1.44	ND	2.1% / 6.2%	TraM-like conjugal transfer protein
Cj0456c	*cj0456c*	Putative Uncharacterized Protein	-1.65	-1.47	0.6% / 1.6%	Clan AA aspartic protease-like protein
Cj0496	*cj0496*	**Putative Uncharacterized Protein**	-1.63	ND	8.1% / 1.6%	YkgJ-like zinc/iron-chelating protein, cysteine cluster protein
Cj0510c	*cj0510c*	**Putative Uncharacterized Protein**	-2.66	ND	4.2% / 5.2%	Metal-sensitive transcriptional repressor
Cj0519	*cj0519*	**Putative Rhodanese-Like Domain Protein**	-1.44	-1.52	2.9% / 2.9%	Sulfur transferase-like protein
Cj0524	*cj0524*	Putative Uncharacterized Protein	-4.56	-4.23	0.0% / 0.9%	No known functional associations
Cj0547	*flaG*	Flagellar Protein	-1.50	-1.78	0.0% / 5.0%	Flagellar biosynthesis-like protein FlaG
Cj0553	*cj0553*	Putative Integral Membrane Protein	-2.47	ND	1.3% / 4.7%	DUF979-containing protein
Cj0556	*cj0556*	Putative Amidohydrolase Family Protein	-2.82	-5.41	1.5% / 1.1%	Metal-dependent hydrolase
Cj0561c	*cj0561c*	Putative Periplasmic Protein	-1.73	-1.85	0.3% / 0.3%	DUF2860-containing protein
Cj0586	*ligA*	DNA Ligase (Polydeoxyribonucleotide Synthase)	-1.51	-1.60	1.9% / 1.9%	DNA replication and repair protein
Cj0589	*ribF*	Putative Riboflavin Biosynthesis Protein	-1.98	-1.80	1.4% / 1.1%	Riboflavin kinase-like, FMN adenyltransferase-like protein
Cj0600	*cj0600*	**Putative Uncharacterized Protein**	-1.92	ND	2.1% / 1.7%	D-Cys desulfhydrase family protein
Cj0601c	*cj0601c*	Transporter	-1.78	ND	1.3% / 2.5%	Sodium:neurotransmitter family protein
Cj0602c	*cj0602c*	**MOSC Domain-Containing Protein**	-1.68	ND	1.8% / 1.4%	Molybdenum cofactor sulfurase
Cj0604	*cj0604*	Putative Polyphosphate Kinase	-1.49	-1.75	1.0% / 1.7%	ATP/GTP generating protein
Cj0616	*pstB*	Phosphate Import ATP-Binding Protein PstB	-1.42	-1.57	1.6% / 3.3%	ABC superfamily ATP-binding protein

Cj0627	*hypA*	Hydrogenase Maturation Factor HypA	-1.56	ND	6.1% / 2.6%	Metallochaperone-like nickel-dependent hydrogenase
Cj0652	*pbpC*	Penicillin-Binding Protein	-1.53	-1.34	0.5% / 1.7%	Peptidoglycan biosynthesis protein, cell shape rod determinant
Cj0667	*cj0667*	**Putative S4 Domain Protein**	-1.95	-3.02	1.2% / 2.5%	RNA-binding methylase, SAM-dependent
Cj0677	*kdpB*	Potassium-Transporting ATPase ATP-Binding Subunit KdpB	-3.86	-19.1	0.9% / 4.1%	Possible pseudogene, potassium-uptake protein
Cj0679	*kdpD*	Truncated KdpD Protein	-4.59	-4.63	0.5% / 1.5%	Possible pseudogene, potassium-uptake protein
Cj0685c	*cipA*	Invasion Protein CipA	-2.62	-4.14	2.0% / 2.0%	Flagellar modification protein
Cj0699c	*glnA*	Glutamine Synthase	-1.41	-1.28	1.9% / 2.5%	Amino acid (Gln) biosynthesis
Cj0703	*cj0703*	Putative Uncharacterized Protein	-1.41	-1.28	0.6% / 5.1%	DUF3972-containing protein
Cj0762c	*aspB*	**Aspartate Aminotransferase**	-1.37	-3.28	1.8% / 2.3%	PLP-dependent, amino acid metabolism protein
Cj0781	*napG*	**Putative Ferredoxin**	-1.45	-1.52	7.3% / 0.8%	Fe-S cluster ferredoxin protein
Cj0782	*napH*	**Putative Ferredoxin**	-2.18	ND	5.0% / 1.9%	Fe-S cluster ferredoxin protein
Cj0788	*cj0788*	**Putative Uncharacterized Protein**	-1.64	ND	0.6% / 3.1%	2-oxoglutarate:acceptor oxidoreductase-like protein
Cj0789	*cca*	Putative Multifunctional Cca Protein	-1.43	ND	1.1% / 1.9%	tRNA nucleotidyltransferase-like protein
Cj0822	*dfp*	**Coenzyme A Biosynthesis Bifunctional Protein CoaBC**	-1.65	-1.96	2.3% / 1.8%	CoA biosynthesis, Cys-dependent flavoprotein
Cj0826	*cj0826*	Putative Integral Membrane Protein	-1.48	ND	0.9% / 3.2%	LptF/LptG-like polysaccharide export protein
Cj0828c	*ilvA*	Threonine Dehydratase Biosynthetic	-1.63	-1.48	0.5% / 2.5%	PLP-dependent amino acid metabolism enzyme
Cj0848c	*cj0848c*	Putative Uncharacterized Protein	-1.43	ND	0.0% / 3.2%	FlhB flagellar T3SS-like flagellar export protein
Cj0849c	*cj0849c*	Putative Uncharacterized Protein	-1.38	-1.57	0.0% / 1.4%	Flagellar hook FlgK-like protein
Cj0854c	*cj0854c*	**Putative Periplasmic Protein**	-1.78	ND	1.7% / 4.3%	Cytochrome *c*-like protein
Cj0859c	*cj0859c*	Putative Uncharacterized Protein	-1.78	ND	0.0% / 1.4%	DUF2730-containing protein
Cj0861c	*pabA*	Para-Aminobenzoate Synthase Glutamine Amidotransferase Component II	-1.87	ND	2.7% / 3.2%	Folate biosynthesis-associated enzyme
Cj0864	*cj0864*	**Putative Periplasmic Protein**	-4.30	ND	0.8% / 1.7%	Thioredoxin-like thiol:disulfide interchange protein
Cj0865	*dsbI*	**Putative Protein-Disulfide Oxidoreductase DsbI**	-1.98	ND	4.5% / 3.8%	Thioredoxin-like thiol:disulfide interchange protein
Cj0872	*dsbA*	**Putative Uncharacterized Protein DsbA**	-1.51	-1.96	1.4% / 2.8%	Thioredoxin-like thiol:disulfide interchange protein DsbA
Cj0874c	*cj0874c*	**Putative Cytochrome *c***	-3.64	-1.86	3.2% / 3.8%	Di-haem-type cytochrome *c*-like protein
Cj0876	*cj0876*	**Putative Periplasmic Protein**	-3.28	ND	3.2% / 3.2%	Cytochrome *c*-like thiosulfate dehydrogenase / tetrathionate reductase TsdA-like protein
Cj0888c	*cj0888c*	ABC Transport System ATP-Binding Protein	-1.46	-2.04	0.8% / 1.4%	No known functional specificity
Cj0890c	*cj0890c*	Putative Sensory Transduction Transcriptional Regulator	-1.42	-1.42	1.8% / 0.5%	CheY-like DNA-binding response regulator
Cj0892c	*cj0892c*	Putative Periplasmic Protein	-1.71	-2.27	0.6% / 1.2%	No known functional associations
Cj0908	*cj0908*	**Putative Periplasmic Protein**	-2.47	ND	1.9% / 1.9%	2Fe-2S cluster-containing protein
Cj0910	*cj0910*	Putative Periplasmic Protein	-2.34	-1.73	2.0% / 3.3%	No known functional associations
Cj0911	*cj0911*	**Putative Periplasmic Protein**	-1.88	-1.88	1.1% / 1.6%	Thioredoxin-like protein
Cj0935c	*cj0935c*	Putative Sodium:Amino Acid Symporter Family Protein	-1.80	ND	0.9% / 3.6%	Sodium:neurotransmitter symport-like protein
Cj0944c	*cj0944c*	Putative Periplasmic Protein	-1.67	-1.93	0.0% / 1.2%	Flagellar directional FliL-like protein
Cj0951c	*cj0951c*	**Putative MCP-Domain Signal Transduction Protein**	-3.04	-13.01	1.3% / 2.7%	MCP, formate chemotaxis, invasion-associated protein
Cj0952c	*cj0952c*	**Putative HAMP-Containing Membrane Protein**	-4.39	-6.20	1.7% / 4.2%	MCP, formate chemotaxis, invasion-associated protein
Cj0953c	*purH*	Bifunctional Purine Biosynthesis Protein PurH	-1.71	-1.76	1.0% / 2.5%	Phosphoribosylaminoimidazolecarboxamide formyltransferase / IMP cyclohydrolase

Cj0954c	*cj0954c*	Putative DnaJ-Like Protein	-1.52	-2.00	0.0% / 2.3%	Co-chaperone DjlA/TerB tellurium resistance-like protein
Cj0955	*purL*	Phosphoribosylformylglycinamidine Synthase 2	-1.56	-1.83	1.9% / 3.3%	Purine biosynthesis PurL/PurM-like protein
Cj0956c	*mnmE*	tRNA Modification GTPase MnmE	-1.53	-1.89	0.9% / 1.6%	TruE/MnmE tRNA methyltransferase-like protein
Cj0957c	*cj0957c*	Putative Uncharacterized Protein	-1.54	-1.58	0.7% / 1.8%	Similar to *H. pylori* protein that inhibits secretion of specific proteins
Cj0958c	*yidC*	Membrane Protein Insertase YidC	-1.58	-1.68	0.4% / 3.8%	Membrane protein insertion / folding protein
Cj0963	*cj0963*	Putative Uncharacterized Protein	-1.56	ND	3.5% / 2.0%	Uracil DNA glycosylase, DNA repair enzyme-like protein
Cj0976	*cmoB*	**tRNA U34 Carboxymethyltransferase**	-1.52	-2.32	1.7% / 2.1%	SAM-dependent enzyme
Cj0991c	*cj0991c*	**Putative Oxidoreductase Ferredoxin-Type Electron Transport Protein**	-1.37	-1.55	4.5% / 2.9%	4Fe-4S type ferredoxin; Cys rich domain protein
Cj1012c	*cj1012c*	**Putative Membrane Protein**	-4.00	ND	0.7% / 2.0%	HemX-like membrane uroporphorphyrin-III *C*-methyltransferase
Cj1013c	*cj1013c*	**Putative Cytochrome c Biogenesis Protein**	-1.55	-1.47	0.6% / 3.0%	CcmC/CcmF cytochrome assembly-like protein
Cj1026c	*cj1026c*	Putative Lipoprotein	-1.71	-2.18	0.6% / 2.9%	FlgP motility factor-like protein LPP20
Cj1039	*murG*	UDP-*N*-Acetylglucosamine--*N*-Acetylmuramyl-(Pentapeptide) Pyrophosphoryl-Undecaprenol *N*-Acetylglucosamine Transferase	-1.49	-2.49	2.0% / 1.5%	Peptidoglycan biosynthesis-associated enzyme
Cj1049c	*cj1049c*	Putative LysE Family Transporter Protein	-1.56	ND	0.5% / 3.5%	Amino acid exporter LeuE type (Leu/Thr/Arg exporter)
Cj1057c	*cj1057c*	Exodeoxyribonuclease 7 Small Subunit	-1.66	ND	0.0% / 1.8%	Exonuclease VII family protein
Cj1088c	*folC*	Folylpolyglutamate Synthase / Dihydrofolate Synthase	-1.65	-1.94	1.5% / 3.0%	Bifunctional Mur-like peptidoglycan enzyme; folate biosynthesis
Cj1094c	*cj1094c*	Putative Preprotein Translocase Protein	-1.44	ND	1.1% / 2.2%	Sec translocon YajC-like protein translocase
Cj1097	*sstT*	Serine/Threonine Transporter SstT (Na+/Serine-Threonine Symporter)	-2.28	ND	2.2% / 1.7%	Amino acid symporter-like protein
Cj1099	*cj1099*	Peptidase	-1.43	-1.43	1.2% / 2.1%	Oligopeptidase F-like protein
Cj1100	*cj1100*	Hypothetical Protein Cj1100	-1.42	ND	0.7% / 1.4%	No known functional associations
Cj1101	*cj1101*	ATP-Dependent DNA Helicase	-1.81	-1.96	1.3% / 1.3%	UvrD-like helicase ATP-binding protein
Cj1102	*truB*	**tRNA Pseudouridine Synthase B**	-2.04	-7.55	1.5% / 1.1%	Cys-dependent ferredoxin-like fold-containing protein
Cj1107	*clpS*	ATP-Dependent Clp Protease Adapter Protein ClpS	-1.56	ND	1.2% / 6.2%	Clp protease adapter-like protein
Cj1117c	*prmA*	**Ribosomal Protein L11 Methyltransferase**	-1.56	NQ	2.5% / 1.1%	SAM-dependent methyltransferase
Cj1132c	*cj1132c*	Putative Uncharacterized Protein	-1.37	-1.45	3.0% / 2.7%	Ribonuclease H family protein PolB-like 3'-5' exonuclease
Cj1155c	*cj1155c*	Putative Cation Transporting ATPase	-1.47	-1.35	2.5% / 2.4%	Heavy metal translocating P-type ATPase-like protein
Cj1176c	*tatA*	Sec-Independent Protein Translocase Protein TatA	-1.41	-1.68	0.0% / 2.5%	Twin arginine translocation system secretion-related protein
Cj1185c	*petB*	**Cytochrome b**	-1.63	-1.64	0.5% / 4.1%	Di-haem-type cytochrome protein
Cj1186c	*petA*	**Ubiquinol-Cytochrome c Reductase Iron-Sulfur Subunit**	-1.47	-1.36	2.4% / 3.6%	2Fe-2S cluster iron-sulfur domain protein
Cj1230	*hspR*	Heat Shock Transcriptional Regulator	-1.44	-1.40	0.0% / 1.6%	MerR –type HTH transcriptional regulator
Cj1262	*racS*	Two-Component Sensor Histidine Kinase RacS	-1.48	-3.32	2.2% / 1.9%	Two-component regulatory protein
Cj1263	*recR*	Recombination Protein RecR	-1.61	-1.61	3.2% / 1.6%	DNA repair-like protein
Cj1302	*cj1302*	Putative HAD Superfamily Phosphatase Subfamily IIIC	-1.51	-1.34	1.0% / 1.3%	Haloacid dehydrogenase superfamily-like protein
Cj1303	*fabH2*	**Putative 3-Oxoacyl-[Acyl Carrier Protein] Synthase III**	-1.58	-2.23	3.1% / 2.5%	Fatty acid biosynthesis, acetyl-CoA-dependent enzyme
Cj1304	*acpP3*	Putative Acyl Carrier Protein	-1.77	ND	1.4% / 5.5%	Fatty acid biosynthesis, acetyl-CoA-dependent enzyme
Cj1306c	*cj1306c*	Putative Uncharacterized Protein	-1.46	-2.00	2.0% / 2.2%	DUF2920-containing protein
Cj1343c	*cj1343c*	Putative Periplasmic Protein	-1.78	-2.78	1.2% / 2.9%	General secretion pathway protein G-like protein
Cj1344c	*gcp*	tRNA N6-Adenosine Threonylcarbamoyltransferase (tRNA Threonylcarbamoyladenosine Biosynthesis Protein TsaD)	-1.51	-1.73	2.1% / 2.7%	*O*-sialoglycoprotein endopeptidase-like protein

Cj1345c	*pgp1*	Peptidoglycan DL-Carboxypeptidase I (PG Peptidase 1)	-5.18	-14.43	0.0% / 1.5%	Peptidoglycan modification, cell shape determining protein
Cj1350	*mobA*	**Molybdenum Cofactor Guanylyltransferase (Molybdopterin-Guanine Dinucleotide Synthase)**	-2.07	NQ	2.1% / 2.1%	Molybdopterin-associated protein
Cj1354	*ceuD*	**Enterochelin Uptake ATP-Binding Protein**	-1.46	ND	0.4% / 3.6%	Iron ABC transport-like protein
Cj1377c	*cj1377c*	**Putative Ferredoxin**	-1.49	-1.51	4.5% / 2.4%	Fe-S cluster protein
Cj1379	*selB*	Putative Selenocysteine-Specific Elongation Factor	-1.50	-1.36	1.8% / 1.5%	Selenocysteine incorporation, translation-associated protein
Cj1388	*cj1388*	Putative Endoribonuclease L-PSP	-2.04	-1.28	0.8% / 0.8%	RidA/YjgF-like protein, PLP-dependent
Cj1392	*metC'*	**Putative Cystathionine Beta-Lyase *N*-Terminus**	-5.58	ND	2.2% / 4.4%	Potential pseudogene, Cys/Met metabolism, PLP-dependent
Cj1393	*metC'*	**Putative Cystathionine Beta-Lyase**	-3.99	-18.44	1.1% / 2.0%	Potential pseudogene, Cys/Met metabolism, PLP-dependent
Cj1394	*cj1394*	**Adenylosuccinate Lyase**	-4.38	-20.65	0.7% / 3.3%	Putative fumarate lyase
Cj1398	*feoB*	**$Fe(2^+)$ Transporter FeoB**	-1.54	-2.33	1.3% / 3.1%	Ferrous iron transport protein
Cj1399c	*hydA2*	**Putative Ni/Fe Hydrogenase Small Subunit**	-2.50	-4.03	3.0% / 1.4%	Fe-S cluster, cytochrome c oxidoreductase domain protein
Cj1400c	*fabI*	**Enoyl-[Acyl Carrier Protein] Reductase [NADH]**	-2.08	-2.20	0.7% / 2.9%	Fatty acid biosynthesis, acetyl-CoA-dependent protein
Cj1401c	*tpiA*	Triosephosphate Isomerase	-1.60	ND	2.7% / 0.9%	Gluconeogenesis pathway in *C. jejuni*
Cj1402c	*pgk*	Phosphoglycerate Kinase	-2.46	-2.85	1.2% / 2.5%	Gluconeogenesis pathway in *C. jejuni*
Cj1403c	*gapA*	Glyceraldehyde-3-Phosphate Dehydrogenase	-2.29	-2.31	2.1% / 3.0%	Gluconeogenesis pathway in *C. jejuni*
Cj1406c	*cj1406c*	Putative Periplasmic Protein	-2.69	ND	2.6% / 2.6%	DUF1090-containing protein, YqjC-like protein
Cj1407c	*cj1407c*	Putative Phospho-Sugar Mutase	-1.47	-1.96	1.7% / 2.6%	Phosphomanno/gluco mutase-like protein
Cj1409	*acpS*	**Holo-[Acyl Carrier Protein] Synthase**	-1.73	ND	2.6% / 0.9%	Fatty acid biosynthesis, acetyl-CoA-dependent enzyme
Cj1414c	*kpsC*	**Capsule Polysaccharide Modification Protein**	-1.73	-3.76	1.6% / 2.2%	Sulfotransferase-like capsular modification protein
Cj1417c	*cj1417c*	Gamma-Glutamyl-CDP-Amidate Hydrolase	-1.96	ND	3.0% / 1.5%	Capsular polysaccharide biosynthesis protein
Cj1419c	*cj1419c*	**Putative Methyltransferase**	-1.71	-1.51	1.2% / 2.4%	Putative SAM-dependent methyltransferase
Cj1425c	*hddA*	Putative D-Glycero-D-Manno-Heptose 7-Phosphate Kinase	-1.64	-1.89	0.9% / 1.5%	Capsular polysaccharide biosynthesis protein
Cj1453c	*tilS*	**tRNA(Ile)-Lysidine Synthase**	-1.48	ND	1.9% / 1.9%	tRNA converting enzyme (Met – Ile)
Cj1457c	*truD*	tRNA Pseudouridine Synthase D	-1.54	-1.58	0.8% / 2.2%	tRNA biosynthesis enzyme
Cj1460	*cj1460*	Putative Uncharacterized Protein	-1.65	-1.65	1.6% / 1.6%	No known functional associations
Cj1461	*cj1461*	**Putative DNA Methylase**	-1.93	-1.94	0.6% / 0.6%	SAM-dependent DNA methyltransferase, RimJ-like protein
Cj1465	*flgN*	Flagellar Biosynthesis Protein FlgN	-1.40	-1.98	0.8% / 1.8%	Flagellar export / secretion-like protein
Cj1474c	*ctsD*	Putative Type II Protein Secretion System D Protein	-2.09	-2.68	0.6% / 1.3%	General secretion pathway, MskL-like protein
Cj1483c	*cj1483c*	Putative Lipoprotein	-1.39	-1.64	0.6% / 0.6%	Nitrogen fixation FixH-like protein
Cj1484c	*cj1484c*	Putative Membrane Protein	-1.36	-1.50	0.5% / 1.5%	No known functional associations
Cj1487c	*ccoP*	**Cbb3-Type Cytochrome *c* Oxidase Subunit**	-1.38	-1.43	1.4% / 2.8%	*C*-type cytochrome complex-associated protein
Cj1490c	*ccoN*	**Cb-Type Cytochrome *c* Oxidase Subunit**	-1.41	-1.81	0.2% / 5.9%	*C*-type cytochrome complex-associated protein
Cj1496c	*cj1496c*	Putative Periplasmic Protein	-1.44	-1.42	0.6% / 2.9%	Magnesium transport-like protein
Cj1497c	*cj1497c*	Putative Uncharacterized Protein	-1.78	ND	0.7% / 2.8%	FliJ flagellar export apparatus-like protein
Cj1509c	*fdhC*	**Putative Formate Dehydrogenase Cytochrome B Subunit**	-1.40	-1.45	1.0% / 4.2%	Di-haem-type cytochrome
Cj1514c	*cj1514c*	Putative Uncharacterized Protein	-1.47	NQ	2.1% / 2.5%	DMSO/nitrate reductase chaperone-like protein
Cj1531	*dapF*	Diaminopimelate Epimerase (DAP Epimerase)	-1.45	-1.85	3.6% / 2.4%	Amino acid and peptidoglycan biosynthesis enzyme

Cj1541	*pxpA*	**5-Oxoprolinase Subunit A**	-1.46	-1.35	2.4% / 3.5%	Glutathione metabolism, produces Glu
Cj1543	*cj1543*	**Putative Allophanate Hydrolase Subunit 2**	-1.46	-1.56	1.2% / 2.8%	Carboxyltransferase-like, biotin-dependent urea carboxylase-like
Cj1545c	*cj1545c*	**MdaB Protein Homolog**	-1.64	-1.95	0.5% / 3.6%	Flavodoxin-like protein
Cj1546	*cj1546*	Putative Transcriptional Regulator	-1.70	ND	3.5% / 2.6%	HxlR-like HTH protein transcriptional regulator
Cj1547	*cj1547*	Outer Membrane Lipoprotein Blc	-1.48	ND	1.3% / 2.0%	Lipocalin-like, extracellular hydrophobic ligand-binding protein
Cj1574c	*cj1574c*	Putative Uncharacterized Protein	-1.43	NQ	4.8% / 0.9%	Putative NADH dehydrogenase subunit F-like protein
Cj1587c	*cj1587c*	Putative ABC Transporter	-1.59	-1.98	0.7% / 1.7%	Putative multi-drug ABC transporter permease-like protein
Cj1589	*cj1589*	**Putative Uncharacterized Protein**	-2.39	ND	0.0% / 0.9%	Putative thiolesterase-like protein
Cj1608	*cj1608*	Putative Two-Component Regulator	-1.50	-1.40	1.0% / 1.0%	WalR/CheY-family transcriptional regulator-like protein
Cj1612	*prfA*	Peptide Chain Release Factor 1	-1.39	-1.74	0.0% / 2.3%	Translation termination protein
Cj1622	*ribD*	Riboflavin-Specific Deaminase / Reductase	-2.05	ND	2.1% / 1.8%	Dihydrofolate reductase-like domain family protein
Cj1627	*cj1627*	Putative Uncharacterized Protein	-1.78	-2.41	1.2% / 2.0%	DUF4261-containing protein
Cj1631c	*cj1631c*	Putative Uncharacterized Protein	-1.58	ND	0.0% / 1.7%	DUF3135-containing protein
Cj1635	*rnc*	Ribonuclease III	-1.42	-1.68	0.0% / 1.8%	RNA nuclease, CRISPR-associated protein
Cj1666c	*cj1666c*	**Putative Periplasmic Protein**	-1.72	-3.81	2.8% / 4.1%	DUF411-containing, thioredoxin domain-containing protein
Cj1676	*murB*	UDP-*N*-Acetylenolpyruvoylglucosamine Reductase	-1.52	-1.95	1.6% / 1.6%	Peptidoglycan biosynthesis enzyme
Cj1680c	*cj1680c*	Putative Periplasmic Protein	-1.45	-1.48	0.4% / 2.0%	No known functional associations
Cj1687	*cj1687*	Putative Efflux Protein	-1.63	ND	1.4% / 3.5%	Putative MFS-type transporter
